# Emerging Target Discovery Strategies Drive the Decoding of Therapeutic Power of Natural Products and Further Drug Development: A Case Study of Celastrol

**DOI:** 10.1002/EXP.20240247

**Published:** 2025-04-22

**Authors:** Yanbei Tu, Guiyu Dai, Yanyan Chen, Lihua Tan, Hanqing Liu, Meiwan Chen

**Affiliations:** ^1^ School of Pharmacy Jiangsu University Zhenjiang Jiangsu China; ^2^ State Key Laboratory of Quality Research in Chinese Medicine Institute of Chinese Medical Sciences University of Macau Taipa Macao SAR China; ^3^ School of Medicine Jiangsu University Zhenjiang Jiangsu China

**Keywords:** celastrol, combination therapy, drug delivery, natural product, target identification, target validation

## Abstract

Celastrol (CEL) is a natural pentacyclic triterpenoid demonstrating significant therapeutic properties against various diseases. However, the ambiguity of target information poses a significant challenge in transitioning CEL from a traditional remedy to a modern pharmaceutical agent. Recently, the emerging target discovery approaches of natural products have broadened extensive avenues for uncovering comprehensive target information of CEL and promoting its drug development. Herein, diverse target discovery strategies are overviewed for the pharmacological and toxicological studies of CEL, including chemical proteomics, protein microarray, degradation‐based protein profiling, proteome‐wide label‐free approaches, network pharmacology, target‐based drug screening, multi‐omics analysis, and hypothesis‐driven target confirmation. Dozens of CEL targets have been identified, which significantly suggests that CEL functions as a multi‐target therapeutic agent. Further network interaction analysis and frequency analysis of collected targets reveal that PRDXs, HMGB1, HSP90, STAT3, and PKM2 may serve as key targets for CEL. Additionally, this review highlights the positive role of target discovery in facilitating CEL‐based combination therapy and drug delivery, which is essential for further advancing the clinical applications of CEL. Efforts in CEL target identification not only aid in unraveling the scientific underpinnings of its multiple pharmacological effects but also offer crucial insights for further drug development of CEL‐based drugs.

## Graphical abstract

Using celastrol as a case study, this review summarizes various target discovery strategies for natural products, including chemical proteomics, protein microarray, degradation‐based protein profiling, proteome‐wide label‐free approaches, network pharmacology, target‐based drug screening, and indirect strategies. It emphasizes the critical importance of the target discovery platforms and identified target information in bioactivity discovery, mechanism of action clarification, toxicity assessment, combination therapy, drug delivery, and clinical application of celastrol.

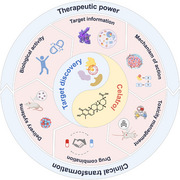

## 1 Introduction

Natural products (NPs) have proven to be a valuable resource in the development of innovative lead compounds owing to their unique structures, diverse pharmacological properties, and novel mechanisms of action [1]. An analysis of small‐molecule drugs approved by the FDA from 1981 to 2019 indicates that 63.1% of them can be linked back to natural origins, either directly sourced or designed based on their pharmacophoric characteristics [2]. This data highlights the indispensable contribution of NPs in drug discovery. Nevertheless, current NP‐based drug discovery still encounters multiple significant challenges, including unfavorable pharmacokinetic properties and complex molecular structures that complicate large‐scale production. In addition, understanding how NPs are involved in regulating biological processes in organisms is critical to moving NPs from the laboratory to clinical drug discovery, which largely relies on the accurate elucidation of targets of bioactive NPs [3]. Target identification is essential for a comprehensive understanding of the therapeutic efficacy and molecular mechanisms of NPs, assessing off‐target adverse effects, and facilitating structure optimization and future drug design [4]. However, the lack of suitable high‐throughput target identification methods in the past has left the targets of most NPs unelucidated. The ambiguity of the targets substantially impedes both the drug discovery process and the clinical application of NPs. Encouragingly, the field of target identification of NPs is undergoing a significant transformation, driven by advancements in cutting‐edge techniques such as proteomics, data science, and the integration of diverse scientific disciplines. The advent of new theories and technologies markedly accelerates the identification of NP targets, reduces time and financial costs, and enhances researchers' comprehension of how NPs elicit their therapeutic or adverse effects. Emerging target discovery approaches are providing new opportunities and unlimited possibilities for NP‐based drug discovery.

**FIGURE 1:**
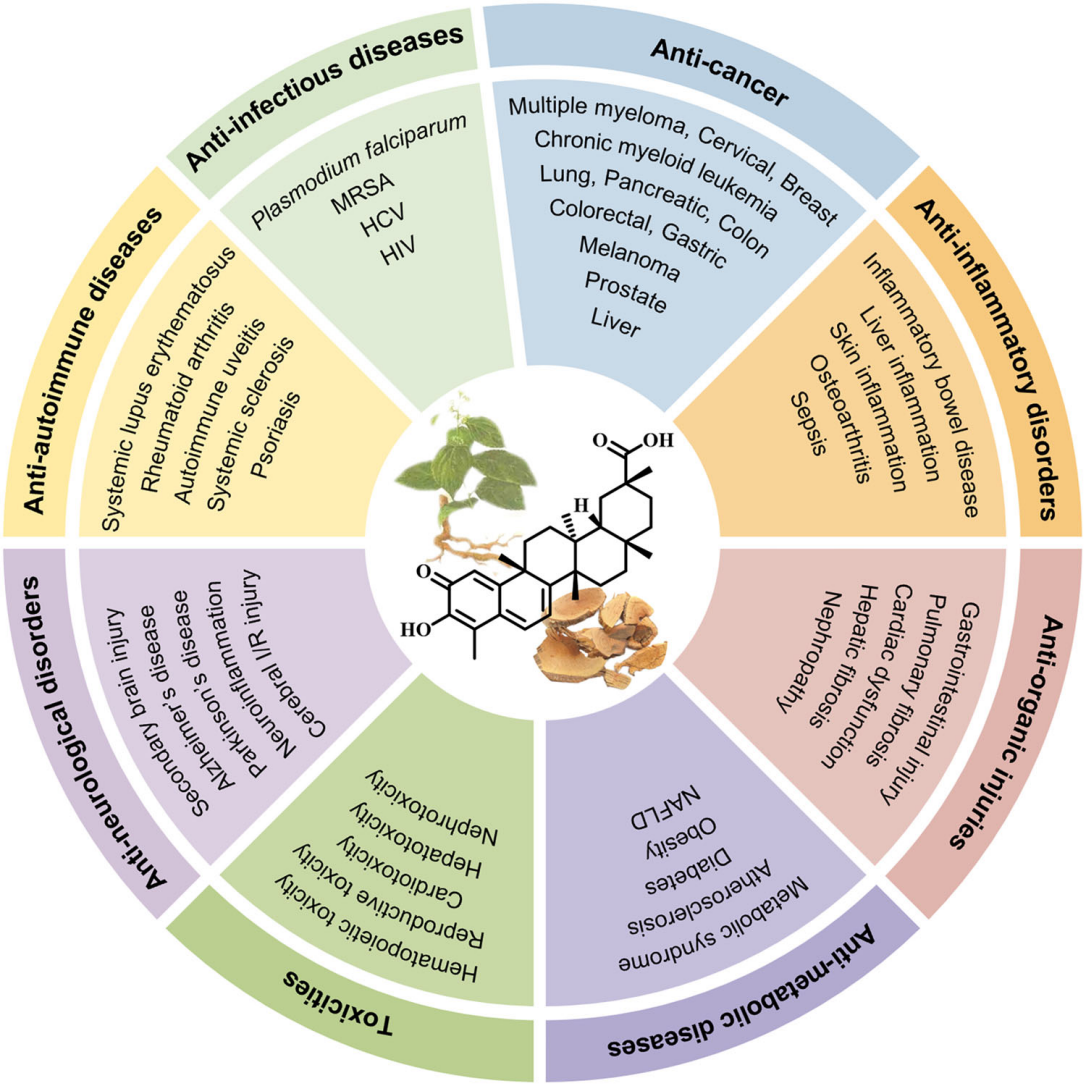
Summary of the pharmacological effects of CEL, including anti‐cancer, anti‐inflammatory disorders, anti‐metabolic diseases, anti‐autoimmune diseases, anti‐neurological disorders, anti‐organic injuries, anti‐infectious diseases, and toxicities.

Celastrol (CEL), also known as tripterine, is a natural friedelane‐type pentacyclic triterpenoid with a molecular formula of C_29_H_38_O_4_ and a molecular weight of 450.61 g/mol, identified from *Tripterygium wilfordii* Hook. F. In 2007, CEL was identified as one of the top five promising NPs for transforming traditional medicines into modern drugs in the journal *Cell* [5]. By 2015, CEL was reported as an effective leptin sensitizer with beneficial weight loss effects [6], thus bringing this traditional Chinese medicinal ingredient into the research spotlight. Numerous studies have demonstrated the favorable pharmacological properties of CEL and its significant potential for clinical use in treating various diseases. For example, CEL has been identified as the primary active component in *Tripterygium wilfordii* (known as “Thunder of God Vine”), a traditional Chinese medicinal herb used for the treatment of autoimmune conditions such as rheumatoid arthritis (RA) [7]. CEL has shown promising potential in treating a range of malignant tumors, including lung, liver, colon, colorectal, gastric, pancreatic, prostate, breast, cervical, ovarian, nasopharynx cancer, and melanoma, through diverse mechanisms of action [8]. In addition, extensive studies have demonstrated the remarkable efficacy of CEL in addressing inflammatory disorders, metabolic diseases, neurological disorders, organic injuries, and infectious diseases (Figure 1) [8, 9].

It is important to highlight that, despite the increasing volume of research regarding the pharmacological properties of CEL, earlier investigations have primarily concentrated on its effects on specific phenotypes or signaling pathways, which limited the understanding of its targets of action. The lack of clear target information presents a considerable obstacle in the process of converting CEL from a traditional remedy into a modern pharmaceutical agent. Recent advancements in drug target identification technologies, such as chemical proteomics, biophysical proteomics, protein microarrays, and network pharmacology, offer promise in uncovering the direct targets of CEL responsible for its pharmacological or toxic effects [3, 4, 10]. While some reviews have discussed the pharmacological activities and mechanisms of CEL [8, 9], there is still a lack of summarizing the various target discovery strategies and reported target information for CEL, as well as utilizing target information to guide pharmacological studies, combination therapy, and drug delivery. Therefore, this review aims to discuss the relationship between the emerging target discovery approaches and pharmacological studies of CEL, using the targets of CEL as a link point. We also summarize the molecular mechanisms of CEL for the treatment of various diseases based on its reported targets. Furthermore, we discuss the beneficial impact of target discovery in facilitating CEL‐based combination therapy and drug delivery while also discussing the existing challenges and perspectives for CEL studies. The mapping of target profiles, driven by target discovery methodologies, will bring a new dawn for CEL‐based drug development.

## 2 Identification of Pharmacological Targets of CEL Based on Different Target Discovery Strategies

Small‐molecule pharmaceuticals typically achieve their intended therapeutic effects by interacting with large biological molecules, primarily proteins, within organisms, a principle that also applies to bioactive NPs [11]. Consequently, the identification of protein targets (referred to “target deconvolution”) and the assessment of the binding extent of drug‐target (referred to “target engagement”) are crucial steps in the discovery and development of novel NP‐based drugs. Presently, direct target identification strategies, including chemical proteomics, protein microarrays, and biophysical proteomics, have been predominant in uncovering targets of NPs [10, 12]. Nevertheless, indirect strategies such as multi‐omics analysis and hypothesis‐driven target confirmation continue to hold a significant position in the target discovery of NPs [12, 13]. Furthermore, virtual screening, network pharmacology, bioinformatics, and artificial intelligence (AI), which rely on virtual technologies rather than real experimental validation, also contribute significantly to the target identification of NPs [14]. Recent years have witnessed the successful target identification of numerous bioactive NPs using these strategies, leading to a deep elucidation of the relationship between NP drugs, targets, molecular mechanisms, and phenotypes. Among these, CEL stands out as one of the extensively studied NPs due to its diverse activities, unique structure, and significant potential for translation to clinical drugs. Therefore, it is imperative to comprehensively review and discuss the application of various target discovery approaches in investigating the mechanisms of action of NPs, using CEL as a representative NP, to advance the research and development of NP‐based drugs (Table 1). In addition, the advantages and disadvantages of each target discovery method are summarized in Table 2, with a view to providing meaningful guidance on the selection of appropriate methodologies for target identification of NPs.

**TABLE 1:** The reported target information of CEL using diverse target discovery approaches.

Target discovery strategies	Target name	Target full name	Sample types	Target validation methods	Binding sites with target	Bioactivity	Ref.
Chemical proteomics (ABPP)	HMGB1	High mobility group protein B1	Primary rat cortical neurons	Pull‐down, IF, CETSA	Covalent bonds (Cys 23/45/106)	Anti‐cerebral I/R injury	[17b]
Chemical proteomics (ABPP)	HnRNPA1	Heterogeneous nuclear ribonucleoprotein A1	BV2 cells	Pull‐down, CETSA, SPR	NT	Anti‐comorbid obesity and depression	[19]
Chemical proteomics (ABPP)	PKM2, HMGB1	Pyruvate kinase M2, high mobility group protein B1, L‐lactate dehydrogenase A chain	LPS‐induced RAW264.7 cells	Pull‐down, IF, CETSA, SPR, MD, TK, UV–vis	Covalent bonds (Cys424 of PKM2; Cys106 of HMGB1)	Anti‐sepsis	[17e]
Chemical proteomics (ABPP)	PRDXs (1,2,4,6), HO‐1	Peroxiredoxins, Heme oxygenase 1	LX‐2 cells	Pull‐down, IF, CETSA, UV–vis, EA, MD, TK	Covalent bonds with Cys	Anti‐hepatic fibrosis	[17a]
Chemical proteomics (ABPP)	Multi‐targets (TUBB, FASN, PRDX1, HSP90β…)	Tubulin alpha‐1B chain, Fatty acid synthase, Peroxiredoxin‐1, Heat shock protein HSP 90‐beta	HCT116 cells	Pull‐down	NT	Anti‐cancer (colon cancer)	[20]
Chemical proteomics (ABPP)	CAND1	Cullin‐associated NEDD8‐dissociated protein 1	Pulmonary fibroblast lysates	Pull‐down, CETSA, IF, MD, TK	Covalent bond (Cys264)	Anti‐pulmonary fibrosis	[17c]
Chemical proteomics (ABPP)	COMT	Catechol O‐methyltransferase	HeLa S3 cells	LC‐MS, labeling assay, IF, EA	Covalent bond (Cys157)	Anti‐neurological disorders	[22]
Chemical proteomics (ABPP)	Nedd4	E3 ubiquitin‐protein ligase NEDD4	Astrocytes	Pull down, CETSA, IF, TK	NT	Anti‐cerebral I/R injury	[17d]
Chemical proteomics (ABPP)	HSP60	Heat shock protein 60	Pulmonary fibroblasts	SPR, CESTA, TK	NT	Anti‐pulmonary fibrosis	[21]
Chemical proteomics (ABPP)	VDAC2	Voltage‐dependent anion‐selective channel protein 2	HepG2 and SKhep1 cells	Pull‐down, CETSA, IF, MD	Covalent bond (Cys13)	Anti‐cancer (hepatocellular carcinoma)	[17f]
Chemical proteomics (ABPP)	PDIA3	Protein disulfide‐isomerase A3	BV2 cell lysates	Labeling assay, SPR	NT	Anti‐neuroinflammation	[23]
Chemical proteomics (ABPP)	PfSpdsyn, PfEGF1‐α	Spermidine synthase, *P. falciparum* elongation factor 1‐α	Parasites	Pull‐down, CETSA, IF, UV‐vis, BLI, MD	Covalent bonds (Cys165/266)	Antimalarial	[24]
Chemical proteomics (ABPP)	HMGB1	High mobility group protein B1	COV434 cells	Pull‐down, CETSA, IF, labeling assay, overexpression	Covalent bond (Cys106)	Reproductive system toxicity	[65]
Chemical proteomics (ABPP)	VDAC1, PC, PKM2, FASN	Voltage‐dependent anion‐selective channel protein 1, Pyruvate carboxylase, Fatty acid synthase, Fatty acid synthase	HK‐2 cells	Pull‐down, CETSA, IF, MD	Covalent bonds with Cys	Nephrotoxicity	[66]
Chemical proteomics (ABPP, IA‐yne)	Multiple targets (GSTO1, PDIs…)	Glutathione S‐transferase omega 1, Protein disulfide isomerases	HeLa cells	Pull‐down, UV–vis, EA	Covalent bond (NT)	Anti‐cancer (cervical cancer)	[25]
Chemical proteomics (CCCP)	Annexin II, eEF1A, β‐tubulin	Annexin A2, Elongation factor 1‐alpha 1, Tubulin beta chain	PANC‐1 cell lysates	Pull‐down	Covalent bonds with Cys	NT	[18]
Chemical proteomics (CCCP)	PKM2	Pyruvate kinase	RAW264.7 cell lysates	Pull‐down, IF, EA, MD	Covalent bond (Cys31)	Anti‐ NAFLD	[26]
Human proteome microarrays	Shoc2	Leucine‐rich repeat protein SHOC‐2	Protein chips	Pull‐down, TK	NT	Anti‐cancer (colorectal cancer)	[28a]
Human Proteome Microarrays	PRDX2	Peroxiredoxin‐2	Protein chips	Pull‐down, SPR, ITC, EA, MD, TK	Hydrogen bond (Cys172)	Anti‐cancer (gastric cancer)	[28b]
Human Proteome Microarrays	STAT3	Signal transducer and activator of transcription 3	Protein chips	Pull‐down, SPR, ELISA, MD, MDS	NT (Leu207, Gln635, Val637)	Anti‐cardiac dysfunction	[28c]
Human Proteome Microarrays	PNPO	Pyridoxine‐5’‐phosphate oxidase	Protein chips	MST	NT	Anti‐cancer (multiple myeloma)	[29]
Degradation‐based protein profiling (DBPP)	IKKβ, PI3Kα, CHK1	Inhibitor of nuclear factor kappa B kinase subunit beta, Phosphatidylinositol‐4,5‐bisphosphate 3‐kinase catalytic subunit alpha, Checkpoint kinase 1	Jurkat cells	WB, Co‐IP, CETSA, IP‐MS	NT	Anti‐cancer, anti‐inflammation, toxicity	[32]
TPP (or MS‐CETSA)	YY1, HMCES	Yin yang 1, Abasic site processing protein HMCES	K562^T315I^ cell lysates	Pull‐down, BLI, TK, MD	Hydrogen bonds, hydrophobic interactions	Anti‐cancer (chronic myeloid leukemia)	[34]
TRAP	CAP1	Adenylyl cyclase‐associated protein 1	THP‐1 cells	Pull‐down, CETSA, MST, ITC, TK	NT	Anti‐metabolic syndrome	[36]
Network pharmacology	PTEN	Phosphatidylinositol 3,4,5‐trisphosphate 3‐phosphatase and dual‐specificity protein phosphatase PTEN	None	MD	NT	Anti‐nephropathy	[39a]
Network pharmacology	MAPK3, TNF, AKT1	Mitogen‐activated protein kinase 3, Tumor necrosis factor, Serine/threonine kinase 1	None	MD	Predicted hydrogen bonds (Tyr176 and Arg172)	Anti‐diabetic nephropathy	[39b]
Network pharmacology	STAT3	Signal transducer and activator of transcription 3	None	MD	Predicted hydrogen bonds (Asp237, Ser319, Asn485)	Anti‐autoimmune uveitis	[40]
Network pharmacology	mTOR	Serine/threonine‐protein kinase mTOR	None	MD, MDS	Predicted hydrogen bonds (Tyr176 and Arg172)	Anti‐osteoarthritis	[41]
Target‐based drug screening (SPR)	Nur77	Nuclear receptor subfamily 4immunitygroup A member 1	SPR chips	CD spectroscopy, HPLC, MD, TK, knockout mice	Covalent bond (Cys551), hydrogen bonds (Asp499 and Gln547)	Anti‐inflammation	[42]
Target‐based drug screening (NanoBiT)	YAP‐TEAD interaction	Transcriptional coactivator YAP1, Transcriptional enhancer factor TEF‐1	NanoLuc‐YAP/TAZ‐TEAD biosensor	Co‐IP, EA	NT	Anti‐cancer	[43]
Target‐based drug screening (BRET)	COMMD3/8 complex	COMM domain‐containing protein 3 and 8	HEK293 cells	Co‐IP, LC‐MS/MS, MD, mutant mice	Covalent bond (Cys170 on COMMD3)	Anti‐rheumatoid arthritis	[44]
Target‐based drug screening (ELISA)	IL‐2	Interleukin‐2	None	SPR	NT	Anti‐cancer (Melanoma)	[45]
Target‐based drug screening (SCLA)	CB2	Cannabinoid receptor 2	HEK293 cells	Functional assays, MD	Predicted hydrogen bond (Thr114)	Anti‐systemic sclerosis	[46]
Transcriptomics	IL1R1	Interleukin‐1 receptor type 1	Mouse hypothalamus	Knockout mice, IL1R1 antagonist	NT	Anti‐obesity	[49]
Multi‐omics	P5CDH	Delta‐1‐pyrroline‐5‐carboxylate dehydrogenase	MRSA USA300 cells	CETSA, BLI, TK, MD	Hydrogen bonds (Lys205 and Glu208)	Anti‐MRSA	[52]
RNA sequencing and OTTER target analysis	PRDX1	Peroxiredoxin‐1	HEK‐293T cells & Data mining	SPR, pull‐down, EA, LC‐MS, Crystallization	Covalent bond (Cys173)	Anti‐cancer (colorectal cancer)	[54]
Metabolomics	FXR	Farnesoid X receptor	Mouse intestinal organoids	DARTS, MD, EA, knockout mice	Hydrogen bond (Arg331)	Anti‐gastrointestinal injury	[50]
snRNA‐seq	EPAC‐1	cAMP‐activated exchange protein‐1	Brain tissues from mice	Pull‐down, CETSA, ITC, TK	cNMP domain (Lys 237 and Arg 279)	Anti‐secondary brain injury	[51]
Hypothesis‐driven confirmation	HSP90α/β	Heat shock protein 90α/β	None	Pull‐down, ELISA, EA, overexpression	C‐terminal domain	Anti‐cancer (pancreatic cancer)	[56]
Hypothesis‐driven confirmation	HSP90β	Heat shock protein HSP 90‐beta	None	DARTS, ITC, EA, MD	NT (Ala47)	Anti‐HCV	[57]
Hypothesis‐driven confirmation	PTP1B, TCPTP	Tyrosine‐protein phosphatase non‐receptor type 1/2	None	Enzyme kinetic, switch SENSE, NMR, knockout mice	Noncompetitive inhibitor, non‐covalent bond	Anti‐obesity	[58]
Hypothesis‐driven confirmation	STAT3	Signal transducer and activator of transcription 3	None	SPR, MD	Predicted hydrogen bonds	Anti‐cancer (colorectal cancer)	[59]
Hypothesis‐driven confirmation	ChREBP	Carbohydrate response element‐binding protein	None	CETSA, DARTS, MD, KD	NT	Anti‐T2DM	[60]
Hypothesis‐driven confirmation, virtual docking	VAMP7, RAB7	Vesicle‐associated membrane protein 7, Ras‐related protein Rab‐7	None	SPR, EA, overexpression	NT	Anti‐obesity	[61]
Hypothesis‐driven confirmation	PPARγ	Peroxisome proliferator‐activated receptor gamma	SPR chips	PPARγ agonist	NT	Anti‐NAFLD	[62]
Hypothesis‐driven confirmation	IL‐17A	Interleukin‐17A	None	SPR, EA	NT	Anti‐psoriasis	[63]

*Note*: NT, not indicated; ABPP, activity/affinity‐based proteome profiling; CCCP, compound‐centric chemical proteomics; TRAP, target‐responsive accessibility profiling; BRET, bioluminescence resonance energy transfer; ELISA, enzyme‐linked immunosorbent assay; SCLA, split luciferase complementation assay; OTTER, Omics and Text based Target Enrichment and Ranking; SPR: surface plasmon resonance; BLI, biolayer interferometry; ITC, isothermal titration calorimetry; MST, microscale thermophoresis; EA: enzymatic assay; CETSA, cellular thermal shift assay; DARTS, drug affinity responsive target stability; IF, immunofluorescence; IP, immunoprecipitation; TK: target knockdown; MD, molecular docking; MDS, molecular dynamics simulations.

**TABLE 2:** Pros and cons of different target discovery strategies of NPs.

Target discovery strategy	Advantages	Limitations
Chemical proteomics	Proteome‐wide target identification; Unbiased and high‐throughput manner; High specificity and accuracy based on NP probes; Compatible with live/intact cells by using different ABPP probes.	Requires the synthesis of NP probes; Non‐specific binding to off‐target proteins; Chemical modifications may alter the target profile of NPs.
Protein microarrays	High‐throughput target screening (>20,000 proteins); Independence from pathological environment; Effective for the identification of low abundance proteins.	Requires the synthesis of NP probes; Targets identified are not linked to specific phenotypes; Chip preparation is costly and requires high‐quality recombinant proteins.
Degradation‐based protein profiling	Proteome‐wide target identification; Suitable for the identification of targets with moderate or weak affinity for NPs.	Requires the synthesis of PROTAC probe pool; Time‐consuming chemical synthesis; Not applicable to proteins not characterized by ubiquitinated degradation.
Proteome‐wide label‐free approaches	Proteome‐wide target identification; No chemical probe is required; Easy to implement, flexible setup, and versatile formats; Compatible with live/intact cells and tissues.	Difficult to detect proteins that do not show significant changes in biophysical properties after drug binding; Relatively low precision.
Network pharmacology	Easy to implement and low cost; Fits with the multi‐targeting properties of NPs.	Targets are predictive; Target validation is time‐consuming and labor‐intensive; Relatively low precision.
Multi‐omics analysis	Provides a comprehensive view of the drug action network High relevance of putative targets to specific disease treatments	Targets are indirectly inferred rather than directly identified; Requires rigorous target validation.
Target‐based drug screening	The target is fixed; High‐throughput drug screening; High confidence in target‐drug binding.	Drug‐target binding is not linked to specific phenotypes; Accuracy is dependent on drug screening methods.
Hypothesis‐driven confirmation	Easy to implement; Based on existing knowledge and information.	Low throughput target identification; Targets are indirectly inferred rather than directly identified. Accuracy is heavily dependent on pre‐existing studies and hypotheses.

### 2.1 Target Discovery Based on Chemical Proteomics

Chemical proteomics is a rapidly developed multidisciplinary approach integrating synthetic chemistry, cell biology, and mass spectrometry (MS) technology in the past few decades [3a]. This approach utilizes molecular probes to selectively target sub‐proteomes interacting with NPs, followed by quantitative proteomics methods to identify the target proteins of these NPs in an unbiased and high‐throughput manner. Molecular probes serve as the hub component of chemical proteomics technology and typically comprise three essential elements: (1) a reactive group, which can be the molecular backbone of the active NPs, or a specific probe designed to target particular types of proteins or amino acid residues; (2) a linker group, which is used to connect NPs with the reporter moiety and to minimize any interference caused by the reporter moiety on the NPs‐target interaction; and (3) a reporter tag, such as solid‐phase matrix, a fluorescent group, an alkyne or biotin, which is employed for the labeling or subsequent enrichment of the target proteins [3, 12, 15].

**FIGURE 2:**
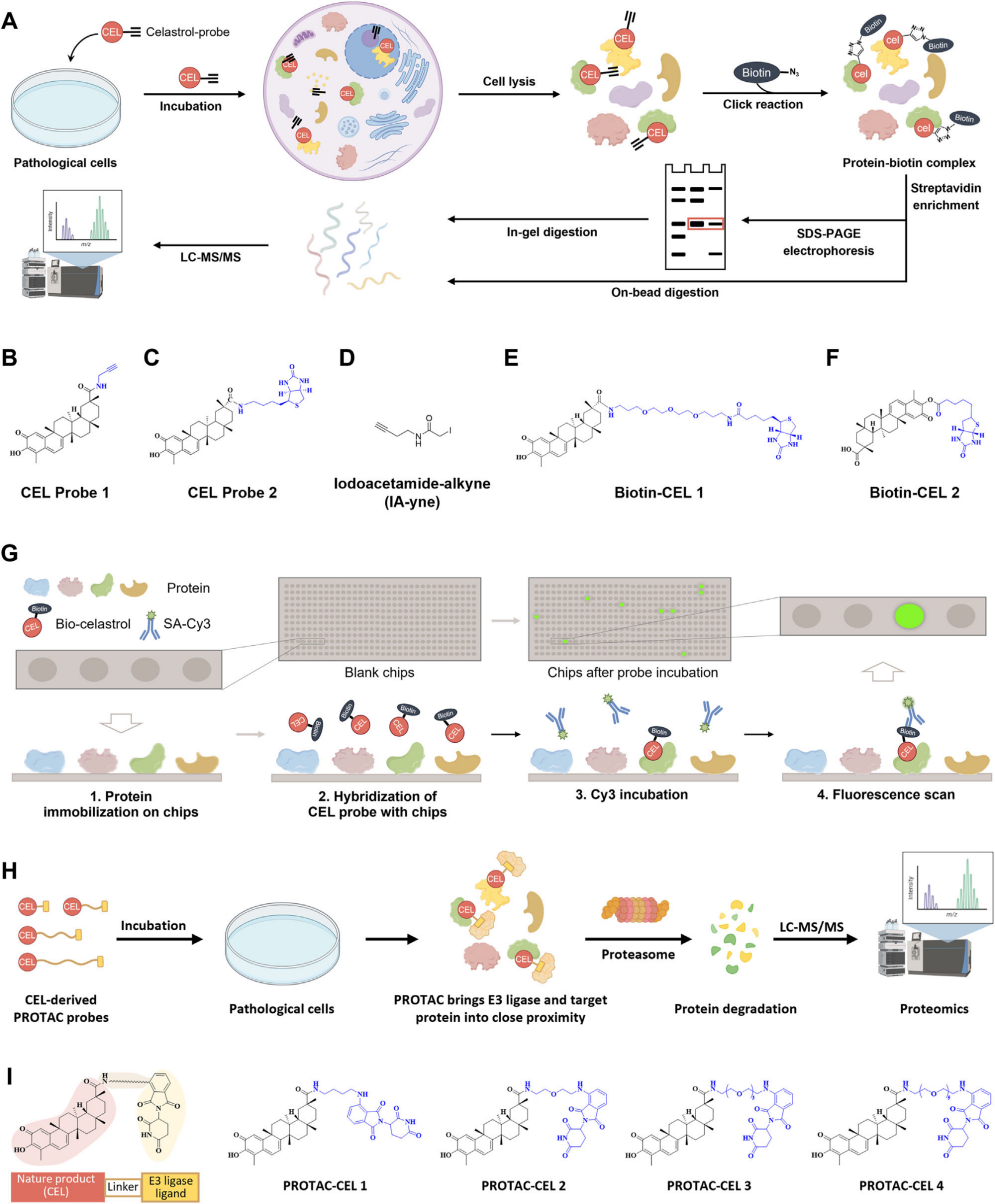
Chemical proteomics, protein microarrays, and degradation‐based protein profiling for the target discovery of CEL. (A) Schematic presentation of chemical proteomics. (B–D) Chemical structures of CEL probes for chemical proteomics. (E,F) Chemical structures of biotinylated probes of CEL for protein microarrays. (G) Schematic presentation of protein microarrays. (H) Schematic presentation of degradation‐based protein profiling. (I) PROTAC probes of CEL.

Based on their different workflows, chemical proteomics approaches can be divided into two categories, namely activity/affinity‐based protein profiling (ABPP/AfBPP) and compound‐centric chemical proteomics (CCCP) [3a]. ABPP is currently the most widely used chemical proteomics technique for the target discovery of NPs using activity‐based probes (ABPs) that are structurally related to NPs. Biotinylated, bioorthogonal, and photoaffinity probes are commonly used ABPs for ABPP methods, which are required to maintain the pharmacological activity of the parent molecules and allow for easy enrichment of the bound protein targets [3, 16]. In a typical ABPP protocol, the active probes are first designed and synthesized based on the structure‐activity relationship (SAR) study of NPs. Next, the probe is incubated with live cells, cell lysates, or tissue homogenates to allow complete binding of target proteins, followed by chemical techniques to enrich target proteins. At last, the enriched proteins are eluted and enzymatically digested to allow for proteome‐wide identification by LC‐MS (Figure 2A). To enhance target identification accuracy, a competitive binding strategy is often employed, where only targets that can be competitively bound by free NPs are considered authentic, thereby minimizing non‐specific binding effects [17]. CCCP is a proteome‐level target identification approach that integrates traditional drug affinity chromatography with modern quantitative proteomics. Unlike ABPP, in the CCCP protocol, the active NP is chemically immobilized as bait on a solid‐phase matrix that serves as a fishing rod (e.g., magnetic or agarose beads), and then the target proteins are pulled down directly from the proteomic pool, such as cell lysates [3, 11]. However, because the immobilization of the NPs makes the “fishing rod” difficult to enter into living cells, CCCP is primarily applicable for target identification within a cell lysate setting.

ABPP‐based chemical proteomics approaches have been widely used for target identification of CEL. The quinone methide moiety of CEL exhibits a high reactivity towards the nucleophilic sulfhydryl groups present on cysteine residues within proteins, resulting in the formation of covalent Michael adducts. These adducts have been shown to impact the functionality of various proteins [8b]. As a result, the phenanthrenequinone methyl ring is recognized as a crucial active functional moiety of CEL. As shown in Table 1, CEL interacts with most of its target proteins by forming covalent bonds with cysteines. Consequently, the design of active probes for CEL typically avoids this reactive group to preserve the biological activity of CEL probes. The carboxylic acid functionality in the CEL structure is considered the most reasonable position for chemical modification due to the simplicity of transformations and weak impact on bioactivities [17, 18]. Presently, the commonly used CEL probes are biotinylated probes and bioorthogonal probes, primarily synthesized through a covalent reaction involving the carboxyl group at the C‐20 position with a biotin or bioorthogonal group (Figure 2B,C). However, the biotinylation of CEL would introduce a larger chemical group on its structure, which may impair biological activity and complicate the synthesis process. In comparison to biotinylated probes, bioorthogonal groups offer the advantage of significantly decreasing the spatial hindrance of active probes. This reduction in spatial resistance enhances the label feasibility of probes within cellular and tissue environments, thereby expanding the scope of potential applications [3, 15]. Zhou et al. [17c] designed seven alkynylated probes with or without photoreactive diazo pyrimidine groups to examine the effect of chemical modifications at the C‐3 and C‐20 positions on CEL activity and found that the structured probes lacking photosensitive groups exhibited notably superior biological activity compared to those containing such groups. Additionally, probes with modifications at the C‐3 position displayed reduced activity in comparison to unmodified probes. This observation may be attributed to the ability of the phenanthrenequinone methyl ring of CEL to establish a covalent bond with the target protein, rendering the photosensitive groups unnecessary for facilitating covalent bond formation in the CEL‐target interaction.

In one study, Liu and colleagues [17b] synthesized CEL probe 1 by linking an alkynyl handle to the carboxyl group of CEL, and the probe showed comparable cytotoxicity and neuroprotective effects to free CEL. Subsequently, in primary rat cortical neurons, an ABPP strategy based on click chemistry reaction was used to reveal that CEL directly targeted HMBG1 without affecting its expression. The covalent bonds (Cys 23/45/106) formed between CEL and HMGB1 significantly contributed to its neuroprotective and anti‐inflammatory effects, thereby alleviating cerebral ischemic‐reperfusion injury (CIRI). In addition, the use of CEL probe 1 revealed multiple targets in different disease conditions, such as HnRNPA1 for the treatment of comorbid obesity and depression [19], PKM2 and HMGB1 for sepsis alleviation in LPS‐induced RAW264.7 cells [17e], PRDXs (1, 2, 4, and 6) and HO‐1 for anti‐hepatic fibrosis effect in LX‐2 cells [17a], multi‐targets (TUBB, FASN, PRDX1, HSP90β…) for anti‐colon cancer activity in HCT116 cells [20], CAND1 [17c] and HSP60 [21] for anti‐pulmonary fibrosis effect in pulmonary fibroblasts, COMT for combating neurological disorders [22], Nedd4 for CIRI alleviation in astrocytes [17d], VDAC2 for anti‐hepatocellular carcinoma activity in HepG2 and SKhep1 cells [17f], PDIA3 for anti‐neuroinflammation action [23], PfSpdsyn and PfEGF1‐α for antimalarial effects in parasites [24]. Taking advantage of the fact that CEL can react with protein thiols covalently and reversibly, Chen et al. [25] used a competitive chemical proteomics approach based on a cysteine‐targeted active probe (IAA, Figure 2D) for the quantitative proteome‐wide analysis of the cellular targets of CEL in human cervical carcinoma HeLa cells, and identified more than 60 target proteins, such as GSTO1 and PDIs.

The use of the CCCP approaches for target discovery of CEL has also been reported. Klaic et al. [18] first synthesized biotinylated CEL, which was subsequently immobilized on neutravidin agarose beads. Using such a “fishing rod,” the researchers have pulled down three protein targets of CEL from pancreatic cancer cell lysates, namely, annexin II, eEF1A, and β‐tubulin. In another study, Fan et al. [26] first synthesized CEL‐ PEG3‐azide and then coupled it to alkyne‐coated agarose resin with a cleavable linker via click chemistry cycloaddition reaction. This CEL‐coated agarose resin was then incubated with lysates from LPS‐stimulated RAW264.7 cells, and PKM2 was identified as the major protein binding to CEL by pull‐down assay. Despite the effectiveness of the CCCP method, its usage is currently not widely due to the well‐established synthesis of bioorthogonal probe (probe 1) for CEL, which offers advantages over immobilized probes in target identification. Collectively, these studies demonstrate that chemical proteomics techniques are very powerful and attractive for target discovery of CEL.

### 2.2 Target Discovery Based on Protein Microarray

A protein microarray is a protein immobilization‐based chemical proteomics strategy to identify target proteins of drugs, which offers a high‐throughput platform for analyzing the interaction of NPs with over 20,000 purified proteins [3, 27]. In this protocol, purified proteins are first immobilized onto miniature high‐density arrays, followed by the hybridization of an NP probe labeled with a reporter tag (e.g., biotin, fluorescent tags, and photochemical tags) with the proteins on the microarrays. Biotinylated probes of NPs are among the most frequently utilized probes for protein microarrays (Figure 2E,F). Subsequently, a fluorescence scanner is used to visualize and quantify the interactions between the NP probe and the proteins immobilized on the chip (Figure 2G) [3, 28]. The labeling of NPs is essential for tracking their physical presence and location as they bind to the protein microarrays. However, the labeling process must not compromise the biological activity of NPs to minimize the generation of false‐positive results. Some well‐established protein microarrays have been developed, including HuProt Human Proteome Microarrays, ProtoArray Human Protein Microarrays, and Human Protein Atlas Protein Fragment Arrays. The choice of which chip to use for research depends on the specific biological application [27b]. Protein microarray offers several benefits. First, it requires a minimal sample size, thereby minimizing sample loss. Second, this method enables the high‐throughput analysis of drug‐target binding interactions across the complete proteome on a microarray, facilitating the identification of all proteins that bind to the drug, whether they are intended targets or unintended off‐targets. Third, the use of purified proteins immobilized on microarrays ensures a consistent abundance of a vast array of proteins, enabling the detection of drug‐target binding interactions that are difficult to capture due to limited protein abundance in the intracellular environment. Given these advantages, recent studies have highlighted the application of protein microarrays in the target discovery for CEL. By using this method and subsequent multilevel target validation, Shoc2, PRDX2, and PNPO were identified as significant targets for its inhibition of colorectal cancer, gastric cancer, and multiple myeloma, respectively [28, 29]. In addition, the target identification results revealed that CEL is directly bound to STAT3 and inhibited its phosphorylation and nuclear translocation, thereby exerting cardioprotective effects [28c].

Protein microarray data present an opportunity to share target information for NPs, such as CEL. This approach directly uses immobilized recombinant proteins to uncover targets, and the screened target proteins can be identified as direct binding targets for NPs. The targets identified by this approach are independent of the pathological environment and thus can provide a very meaningful reference for the exploration of therapeutic targets of NPs in different disease conditions. Nevertheless, it is crucial to note that not all targets identified via protein microarrays necessarily correspond to the targets of NPs that exhibit efficacy in a specific disease state. To pinpoint the authentic target of action for NPs, it is imperative to conduct multiple validations of NP‐target binding within a pathological setting. Additionally, the inclusion of a free biotin control is essential to eliminate potential interference from biotin labeling on NPs, thereby ensuring the precision of target identification. At present, over 20,000 recombinant proteins have been immobilized onto universal human proteomic microarrays, and the advancements in protein microarrays are anticipated to enable larger‐scale and more efficient target screening of NPs in the future.

### 2.3 Target Discovery Based on Degradation‐Based Protein Profiling or Targeted Degradomics

Currently, the accurate identification of target discovery methodologies, represented by APBB and CCCP, relies heavily on the presence of a sufficiently robust affinity between the drug and the target protein. However, many NPs do not bind strongly to the target but still exert their pharmacological effects through the modulation of that target. Regrettably, most of the current target discovery techniques are inadequate for detecting targets characterized by moderate or weak binding affinities with NPs. Proteolysis‐targeting chimeras (PROTACs) represent a novel approach for the degradation of target proteins via the Ubiquitin‐Proteasome System (UPS), which has garnered significant attention in recent years. PROTACs are structurally composed of three components: a ligand for an E3 ubiquitin ligase, a ligand for the target protein, and a specially designed linker that connects the two active ligands, thereby forming a ternary complex [30]. Inspired by the fact that PROTAC molecules often do not require strong affinity to efficiently and specifically degrade target proteins, Wu et al. (2022) introduced a method that integrates PROTAC probes with quantitative proteomics, termed “Targeted Degradomics” [31]. In this protocol, a bifunctional PROTAC probe derived from NPs is employed to achieve ubiquitinated degradation of target proteins by recruiting the targeted proteins to the proximity of the E3 ubiquitin ligase. Subsequently, proteins that exhibit reduced expression levels (degraded proteins) in the proteomics data relative to the control group are identified as binding targets of NPs through differential proteomics analysis and evaluation (Figure 2H,I). Utilizing this innovative technique, MAFF protein was successfully identified as a credible target for lathyrol, a new lathyrane diterpenoid isolated from *Euphorbia lathyrism* [31]. In 2024, Ni et al. developed a multi‐target identification strategy known as “degradation‐based protein profiling (DBPP),” which combines PROTAC technology with quantitative proteomics and immunoprecipitation‐mass spectrometry (IP‐MS). By using the synthesized PROTAC toolbox composed of multiple PROTAC probes derived from CEL, the known targets IKKβ and PI3Kα were successfully identified, together with the new target CHK1 exhibiting a weak binding affinity with CEL [32].

Innovative DBPP or targeted degradomics strategies could well complement existing chemical proteomics technologies, thereby facilitating more effective and thorough target identification of NPs. Both methods require structural modification of the active NPs to create PROTAC molecular probes. Therefore, these methods rely on chemically induced protein‐protein interactions (PPIs) rather than traditional protein‐small molecule interactions [32]. Compared to traditional chemical proteomics methodologies, this approach is highly effective for identifying target proteins with moderate or weak binding affinity, greatly broadening the range of identifiable target proteins. Despite its advantages, DBPP or targeted degradomics strategies present several limitations. As a ternary complex, the design and synthesis of PROTAC molecules are inherently complex. The orientation, type, and length of the linker play a crucial role in determining the selectivity and efficacy of target degradation by PROTAC molecules. Furthermore, the selection of E3 ligases ligand introduces variability in the targeted degradation of proteins. Consequently, proteomic assays utilizing a PROTAC pool, which consists of multiple active PROTAC probes derived from NPs, are necessary to enhance assay accuracy. However, this requirement results in time‐consuming and labor‐intensive processes for chemical synthesis and quantitative proteomic analysis. Currently, the design of PROTAC probes within the DBPP framework is restricted to the E3 ligase ligand CRBN, indicating a need for the exploration of a broader range of ligands, such as VHL, MDM2, and cIAP1, in future research [30]. Additionally, it is important to note that not all instances of protein ubiquitination result in protein degradation, and the DBPP approach is limited in its ability to identify target proteins exhibiting such characteristics. In summary, the advancement of DBPP technology is inextricably linked to the ongoing optimization of PROTAC technology and the iterative design of PROTAC molecules. It is anticipated that the DBPP strategy will play a significant role in the identification of drug targets in the future, thereby facilitating the development of more effective therapeutic agents.

### 2.4 Target Discovery Based on Proteome‐Wide Label‐Free Approaches

**FIGURE 3:**
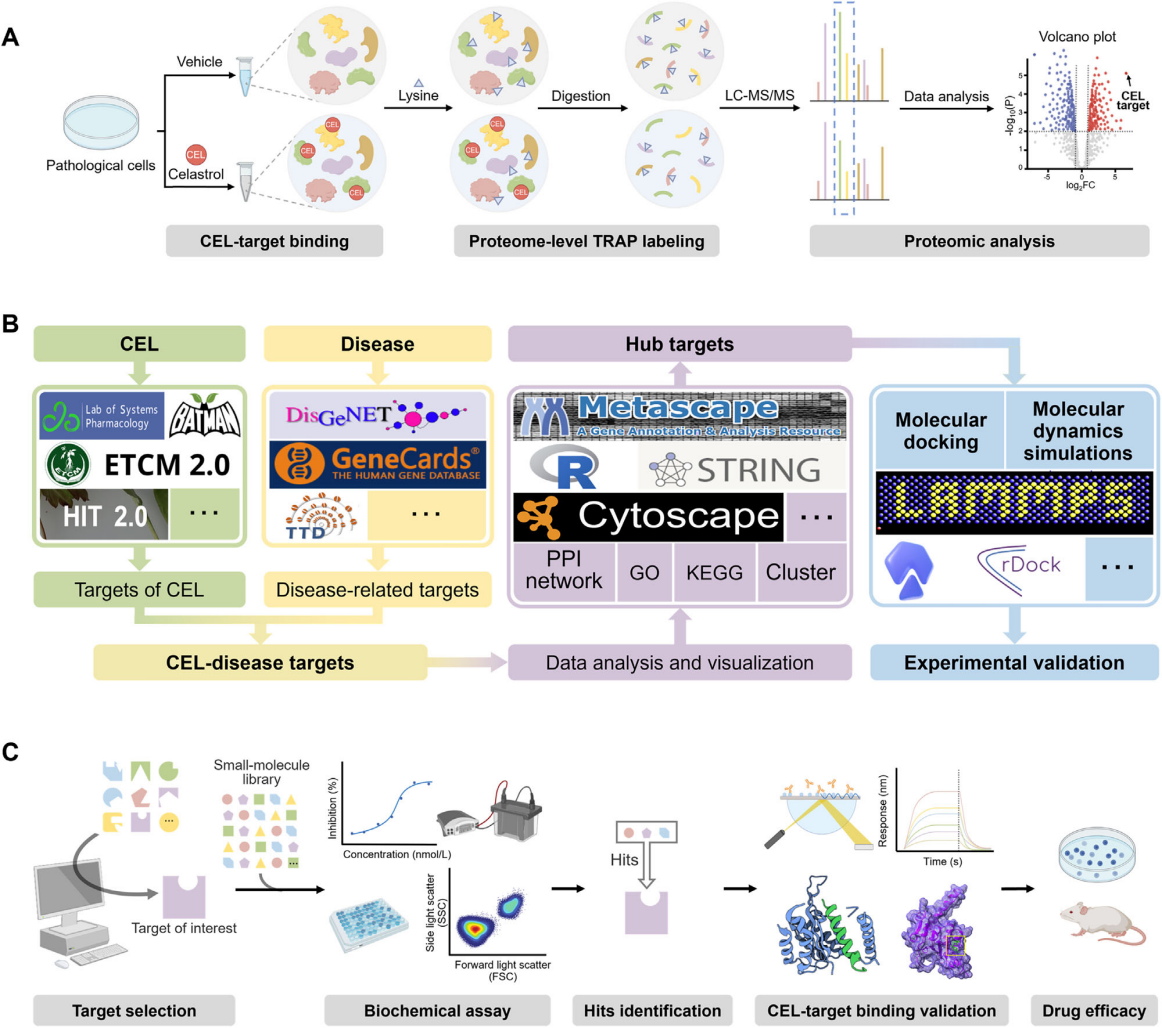
TRAP assay, network pharmacology, and target‐based drug screening for target discovery of CEL. (A) Schematic presentation of TRAP assay. (B) Schematic presentation of network pharmacology. (C) Schematic presentation of target‐based drug screening.

Although advancements in chemical proteomics or protein microarray have significantly improved the identification of targets for NPs, particularly CEL, there are still limitations, such as the necessity to chemically modify the structure of NPs for probe synthesis and the possibility that synthetic probes may not exhibit identical target profiles as the original NPs [3a]. Therefore, the recently emerging label‐free target protein screening approaches have gained considerable attention for identifying NPs targets, including drug affinity responsive target stability (DARTS) method, pulse proteolysis (PP), MS‐cellular thermal shift assay (MS‐CETSA, or named thermal proteome profiling), stability of proteins from rates of oxidation (SPROX) method, solvent‐induced protein precipitation (SIP) method, and chemical denaturation and protein precipitation (CPP) method [10, 33]. These techniques enable target identification of NPs by directly observing specific changes in protein properties upon binding of NPs, such as alterations in protein hydrolysis sensitivity, thermal stability, oxidative stability, and chemical denaturation‐induced stability. A key characteristic of these methods is their ability to identify target proteins of NPs without the need for probe synthesis or chemical modification of NPs, which reduces the difficulty and effort of chemical modification of complex NPs [33a]. However, there are limitations associated with this approach. For instance, drug treatment may not induce significant changes in all biophysical properties of target proteins, potentially leading to the oversight of certain insensitive target proteins. Moreover, alterations in target proteins induced by NPs could affect the biophysical properties of other proteins within the protein complex, resulting in inaccurate target identification [10, 33]. To enhance the precision of identification, it is advisable to complement this approach with chemical proteomics or other methodologies. Unfortunately, the above‐mentioned label‐free strategies for CEL target discovery have been less reported, which may be related to the fact that the design and synthesis of chemical proteomics probes for CEL are already very mature. Recently, by using the MS‐CETSA approach in BCR‐ABL T315I‐mutant K562 cells, YY1 and HMCES have been identified as the key targets of CEL. Through the interaction with these two targeting proteins, CEL could induce DNA damage and cell death to combat drug‐resistant chronic myeloid leukemia [34]. Target‐responsive accessibility profiling (TRAP) served as a novel label‐free technique developed by Tian and colleagues [35]. It characterized the solvent accessibility of proteins through lysine labeling at the whole proteome level and then compared the changes in protein accessibility before and after ligand binding to identify targets of ligands and ligand‐binding regions [35]. Notably, the TRAP method also does not require chemical modification of the drug molecule, making it particularly suitable for target identification of NPs (Figure 3A). In a separate study, Zhu et al. [36] employed the TRAP technique in THP‐1 cells to uncover the binding protein of CEL as CAP1. Their findings demonstrated that CEL bound to CAP1 disrupted the interaction between resistin and CAP1, and effectively attenuated the ensuing inflammatory response, which ultimately ameliorated metabolic syndrome. Collectively, the potential of label‐free approaches for CEL target identification is still worth to be further explored.

### 2.5 Target Discovery Based on Network Pharmacology

Network pharmacology is a methodology rooted in systems biology and bioinformatics that examines the intricate network of interactions involving drugs, protein targets/genes, and diseases within biological systems from a holistic and systemic viewpoint [14, 37]. This feature of network pharmacology is in line with the characteristic of “multi‐component, multi‐target, and multi‐actions” of herbal medicines, particularly Chinese medicines, so when it first emerged, the technology was mainly used for systematic studies on the pharmacological effects, mechanisms of action, safety evaluation of Chinese medicines [38]. Nevertheless, given that many NPs also exhibit pharmacological effects through multiple targets, network pharmacology has more recently been employed in identifying the targets of individual NPs, garnering considerable attention. In a typical network pharmacology protocol, the target information of NPs and specific diseases is initially gathered separately, and then the intersection targets of the two are predicted to be the target group of NPs against a certain disease. Subsequently, a protein–protein interaction (PPI) network of the intersected targets or “NPs‐target‐disease” network is constructed and analyzed using bioinformatics methods, with the core targets in the network identified as candidate targets for subsequent drug‐target binding investigations employing various methodologies (Figure 3B). Through the establishment of an interaction network between CEL and multiple diseases and subsequent network analysis, PTEN, MAPK3, TNF, and AKT1 were identified as core targets for CEL to exert its role in mitigating renal injury and diabetic nephropathy [39], whereas the anti‐autoimmune uveitis effect of CEL was closely related to the modulation of STAT3 [40].

The network pharmacology process is simple and does not require the support of well‐established experimental conditions, and therefore, it consumes relatively inexpensive experimental time and financial costs. While this approach enables researchers to swiftly identify targets of NPs and pinpoint targets of interest, it is essential to recognize that network pharmacology is a predictive rather than an experimental discovery method, and therefore, the results it gives for target discovery may not always be accurate. Consequently, it is crucial to validate the interaction between NPs and candidate targets post‐identification of the core targets through network pharmacology. In addition, the integration of network pharmacology data with actual transcriptomic and proteomic datasets can significantly enhance the precision of target prediction. For instance, Dai et al. [41] combined transcriptomics and network pharmacology analysis to identify the hub target of CEL against osteoarthritis as mTOR. Network pharmacology offers the opportunity to examine the mechanisms of action of NPs against specific diseases from a holistic network perspective, yet current research predominantly concentrates on a limited number of core or individual targets. Therefore, establishing an effective experimental methodology to comprehensively elucidate the overall interaction of target networks and NPs is an area warranting further attention. As an emerging and important tool, the introduction of AI into network pharmacology will facilitate the analysis of big data, deep mining of network relationships, and rapid and accurate target identification. This underscores the pivotal role that AI is poised to play as a catalyst in the future development of network pharmacology technologies.

### 2.6 Target Discovery Based on Target‐Based Drug Screening

High‐throughput drug screening based on a certain target highly correlated with disease mechanisms remains one of the most important tools for novel drug discovery. Unlike approaches such as chemical proteomics and network pharmacology that involve forward target identification from the drug, target‐based drug screening involves the reverse process of identifying potential lead compounds from the target [11, 12, 15]. NP‐based drug discovery can be accelerated by using this method to rapidly find NPs with high affinity to the target from a compound library consisting of thousands of NPs (Figure 3C). Although target‐based drug discovery avoids the difficulties in target identification, it is essential to emphasize that while the screening process identifies candidate NPs that bind to the target, this does not automatically imply the therapeutic effect of candidates against a particular disease. Therefore, whether the candidate has pharmacological activity based on the target regulation needs to be carefully determined in subsequent pharmacological experiments. For instance, in a study conducted by Hu et al. [42], 136 NPs with established anti‐inflammatory properties were screened using a surface plasmon resonance (SPR)‐based assay to pinpoint candidate NPs capable of binding to Nur77. CEL was identified as a potent compound with a *K*_d_ of 0.29 µM, demonstrating the ability to inhibit inflammation and induce autophagy in a Nur77‐dependent manner. Similarly, Nouri et al. [43] established an ultra‐bright NanoLuc biosensor to quantify the YAP‐TEAD interaction of a library of 2688 small molecules, which identified CEL as a novel YAP‐TEAD inhibitor. In another study, Shirai et al. [44] developed a bioluminescence resonance energy transfer (BRET)‐based live‐cell assay system to screen for inhibitors disrupting the formation of COMMD3 and COMMD8 complexes. Through this system, 2555 NPs were screened, leading to the discovery of CEL as a candidate that covalently binds to and dissociates COMMD3/8 complex (IC_50_ = 0.74 µM). CEL plays an important role in the treatment of autoimmune diseases, such as rheumatoid arthritis, by inhibiting humoral and autoimmune responses through the inactivation of COMMD3/8 complexes. Cho et al. [45] evaluated 900 NPs for inhibition of IL‐2/CD25 binding using a competitive ELISA assay and found that CEL selectively blocked IL‐2 and CD25 binding with the greatest potency (IC_50_ = 2.217 µM). CEL was shown to selectively and directly bind to IL‐2 but not CD25, thereby impairing IL‐2 and CD25 binding and exerting anti‐melanoma activity through the mediation of T‐cell responses. In another study, researchers identified CEL as a novel selective CB2 agonist using the luciferase‐based split luciferase complementation assay (SLCA) performed in HEK293T cells, and subsequent experiments revealed the significant therapeutical effect of CEL on systemic sclerosis [46].

### 2.7 Target Discovery Based on Indirect Strategies

Indirect strategies for target discovery are those that infer the targets of action of NPs by analyzing the alteration in omics data or phenotypic changes induced by NPs or by considering established single or multiple signaling pathways and other known information. They do not directly identify the target of NPs but rather revalidate the target inference based on known information [10, 47]. Presently, indirect strategies continue to play a significant role in the process of discovering targets for NPs.

#### 2.7.1 Target Discovery Based on Multi‐Omics Data

**FIGURE 4:**
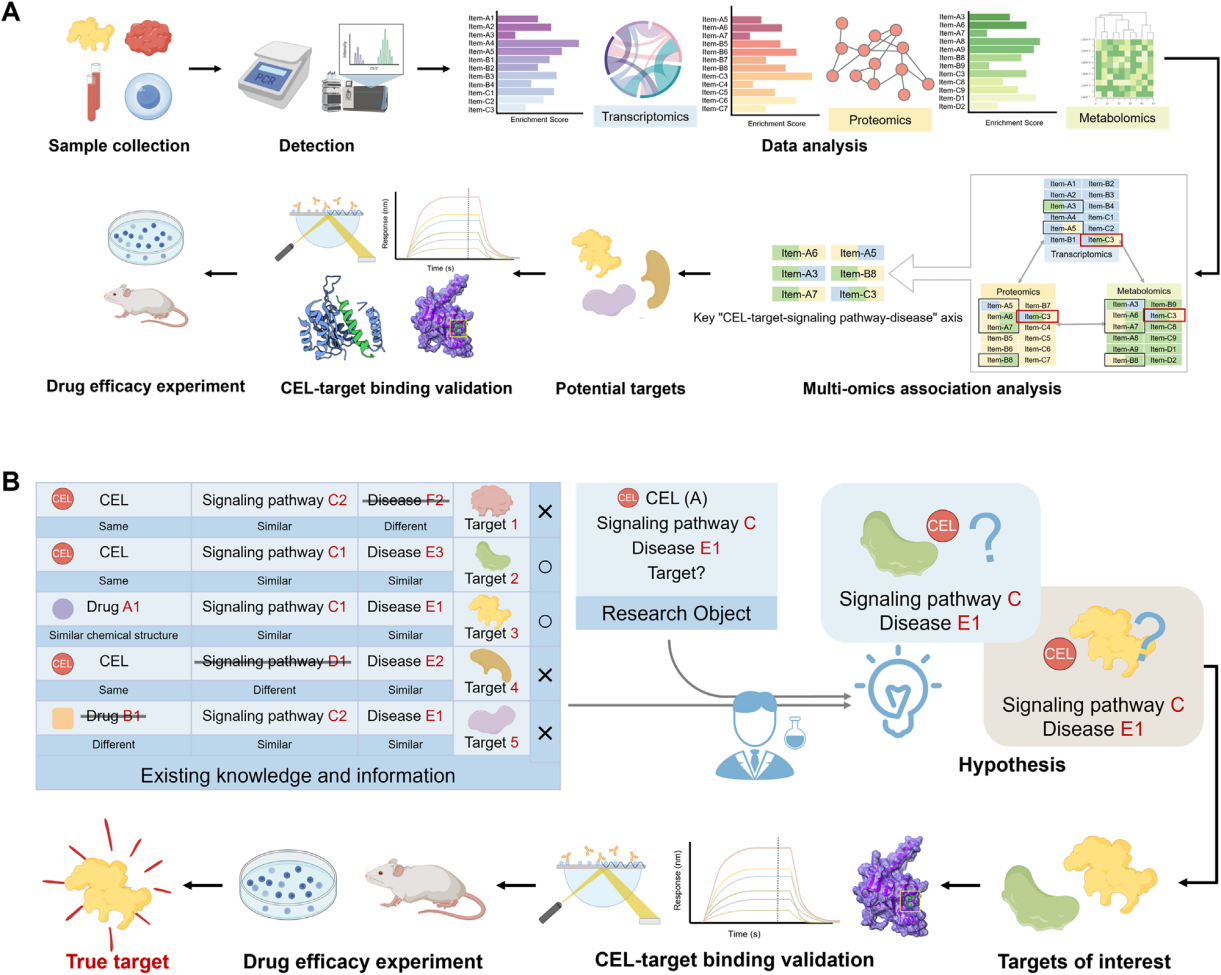
Indirect strategies for target discovery of CEL. (A) Schematic presentation of multi‐omics analysis. (B) Schematic presentation of hypothesis‐driven target confirmation.

Currently, various technologies such as genomics, transcriptomics, proteomics, and metabolomics are advancing rapidly and continuously evolving. Target speculation based on multi‐omics data analysis is a crucial method for inferring the targets of NPs. By observing the global perturbation of genes, mRNA, proteins, or metabolites in pathological cells or tissues induced by NPs, this method speculates the hub targets closely related to pharmacological effects and the biological pathways associated with these targets, which can quickly sort out the “drug‐target‐signaling pathway‐disease” axis and provide meaningful guidance for investigating the mechanisms of action of NPs (Figure 4A) [10, 13, 48]. For example, Feng et al. [49] identified IL1R1 as the target responsible for the anti‐obesity effects of CEL by analyzing transcriptomic data from the hypothalamus of CEL‐treated diet‐induced obese (DIO), lean, and db/db mice. In another study, untargeted metabolomic analysis of plasma and cecum contents from CEL‐treated mice showed that CEL inhibited intestinal FXR signaling, and further studies indicated that CEL was a novel intestinal FXR antagonist [50]. Additionally, single‐cell nucleus RNA sequencing (snRNA‐seq) on mice brain tissues revealed that the cAMP/EPAC‐1 signaling pathway was affected by CEL. Subsequent binding validation showed that CEL bound to cNMP (a structural domain of EPAC‐1) and improved neuronal mitochondrial dysfunction through interaction with EPAC‐1 [51].

Compared to the single‐omics analysis, the effective integration of multi‐omics data is regarded as the future trend of this strategy. In one study, Yuan et al. [52] discovered significant anti‐methicillin‐resistant *Staphylococcus aureus* (MRSA) activity of CEL and proposed P5CDH as its potential antimicrobial target through multi‐omics (transcriptomics, proteomics, and metabolomics) analysis of CEL‐induced MRSA USA300 cells. While omics data have been commonly utilized for unbiased target prediction, pinpointing a specific phenotypic target from numerous genes or proteins remains highly challenging. In recent years, with the continuous development of multi‐omics technologies and their wide application in TCM research, a large amount of global omics data related to herbal medicines and NPs has been accumulated. Therefore, the introduction of AI technologies, such as deep learning methods to realize rapid and accurate mining of massive omics data can provide new opportunities for the analysis of the mechanism of action of herbal medicines and NPs [53]. In one study, Xu and colleagues introduced OTTER (Omics and Text‐based target Enrichment and Ranking), a text‐mining web server tool designed to aid researchers in accurately inferring targets from omics data of query compounds. The researchers analyzed RNA sequencing data from CEL‐treated HEK‐293T cells and used the OTTER tool to successfully identify PRDX1 as a CEL target for regulating ROS in colorectal cancer [54].

Although multi‐omics techniques have been extensively employed to investigate the mechanisms of action of NPs, the vast majority of the omics data generated from these studies are derived from cellular or animal tissue samples rather than clinical samples. This inconsistency may lead to omics perturbation data that fail to accurately reflect the physiological environment, thereby compromising the accuracy of target identification and impeding the translation of NPs into clinical applications. The advent of organoid‐on‐chips technology, which integrates organoid culture with microfluidic chip technology, presents a novel opportunity for advancing multi‐omics research of NPs. Organoids can mimic the three‐dimensional cellular structure of real human organs and can maintain the physiological functions and properties of certain organs in vitro. The microfluidic chip facilitates the simultaneous processing of multiple samples, thereby enhancing the efficiency of drug screening and omics studies [55]. By assessing the impact of NPs on cellular behavior and metabolic processes within organoid microarrays, researchers can acquire omics data that more closely resemble clinical conditions, thus enabling the identification of critical targets and mechanisms underlying the medicinal effects of NPs. This approach also offers the capability to monitor the physiological state of organoids and their responses to drugs in real‐time, providing valuable data for the development of NPs. Personalized precision medicine has emerged as a central focus in contemporary healthcare. By utilizing patient‐derived cells to generate organoids, researchers can conduct personalized drug response studies, thereby enhancing the therapeutic application of NPs in individualized treatment regimens. Collectively, multi‐omics analysis based on organoid‐on‐chips holds significant promise for target identification of NPs, which may enhance the understanding of clinical efficacy, mechanisms of action, and personalized medicine. Nonetheless, there is currently a lack of reports regarding the application of this technology for CEL target discovery.

#### 2.7.2 Target Discovery Based on Hypothesis‐Driven Confirmation

Hypothesis‐driven target identification strategy refers to the use of existing knowledge and information (e.g., identified disease targets or signaling pathways) to infer the target of NPs, and then to directly detect the binding of the NPs to the target to determine whether it is the action target of NPs responsible for a certain bioactivity (Figure 4B). Unlike experimental or predictive target identification methods, this method relies heavily on the researcher's familiarity with the information available in the field and the careful observation of indirect experimental phenomena to improve the accuracy of target discovery. The correctness of the hypothesis in a hypothesis‐driven target identification strategy is gauged by the subsequent evidence supporting drug‐target binding. For example, based on previous reports that CEL regulated the HSP90 pathway and induced heat shock protein translation, the researchers postulated that CEL could function as a novel HSP inhibitor. Subsequent validation experiments strongly supported this hypothesis by demonstrating CEL's disruption of the HSP90‐Cdc37 interaction, leading to an anti‐pancreatic cancer effect [56]. Furthermore, CEL was identified as an inhibitor targeting HSP90β, which inhibited hepatitis C virus translation and associated inflammation [57]. Based on the screen results of protein tyrosine phosphatase inhibitors that have already been reported and on the known relationship between PTP1B and TCPTP and leptin regulation, CEL was inferred and demonstrated that it reversibly bound to and non‐competitively inhibited PTP1B and TCPTP activity to exert a weight loss effect [58]. Additionally, employing this strategy has led to the discovery of other targets of CEL such as target STAT3 for anti‐colorectal cancer activity [59], target ChREBP for anti‐type 2 diabetes effect [60], targets VAMP7 and RAB7 for anti‐obesity activity [61], target PPARγ for anti‐NAFLD effect [62], and target IL‐17A for anti‐psoriasis activity [63]. While the experimental design of this strategy is relatively straightforward and simple to execute, it is heavily dependent on pre‐existing studies and hypotheses, which may constrain the identification of novel targets. Furthermore, the strategy exhibits low throughput and is insufficient for comprehensively elucidating drug‐target interactions in complex diseases. Consequently, it is advisable to exercise caution when employing this approach in future research endeavors.

## 3 Identification of Toxic Targets of CEL Based on Target Discovery Strategies

Although CELs have demonstrated considerable potential for the treatment of various diseases in preclinical investigations, their progression to clinical applications is hindered by numerous challenges. A primary concern is the significant toxic side effects associated with CEL use, which include reproductive toxicity, cardiotoxicity, hepatotoxicity, nephrotoxicity, and hematopoietic system toxicity. The toxicological profiles of CEL have been comprehensively reviewed in several publications [8, 9, 64]. To facilitate the clinical utilization of CEL, it is imperative to minimize these adverse effects and improve beneficial effects, which necessitates a thorough understanding of the mechanisms by which CEL inflicts harm on biological systems. Target discovery methods, which have been evolving in recent years, not only aid in identifying pharmacodynamic targets but also offer valuable insights into the toxicological impacts of NPs.

The ABPP strategy has significant potential for uncovering toxic targets and evaluating the safety profile of CEL. For instance, a study demonstrated that CEL triggered premature ovarian failure and the apoptosis of granulosa cells. Subsequently, researchers employed the ABPP approach to identify that the toxicity of CEL on the female reproductive system was attributed to the direct interaction with HMGB1, which offered crucial insights for assessing the safety of clinical administration of CEL in female patients [65]. Renal dysfunction may occur in patients taking CEL‐related drugs. A combined analysis of ABPP‐based target identification and subsequent metabolomics has effectively identified VDAC1, PC, PKM2, and FASN as potential targets of CEL associated with nephrotoxicity [66]. In addition, CHK1 kinase was identified to be one of the possible toxicity targets of CEL using the DBPP strategy [32]. CEL is a principal active component in tripterygium glycoside tablets (TGT). While TGT has been utilized in the clinical management of arthritis, drug‐induced liver injury (DILI) associated with its administration remains a significant challenge that impedes its extensive clinical application. By integrating clinical multi‐omics data and chemical and target profiling of TGT, the mechanisms of TGT‐caused hepatotoxicity was proposed to be that TGT led to the “iron‐lipid” disturbances in the liver tissues by regulating STAT3‐HAMP‐ACSL4‐LPCAT3 axis [67]. Despite ongoing research efforts to clarify the toxicological effects of CEL, the identification of toxicological targets remains significantly underreported in comparison to pharmacological targets. Future investigations employing diverse target discovery methodologies to delineate the toxicity target profile and further clarify the toxicological mechanisms deserve more attention.

## 4 Cell‐Free, Cell‐Based, and In Silico Target Validation for Target Discovery of CEL

**FIGURE 5:**
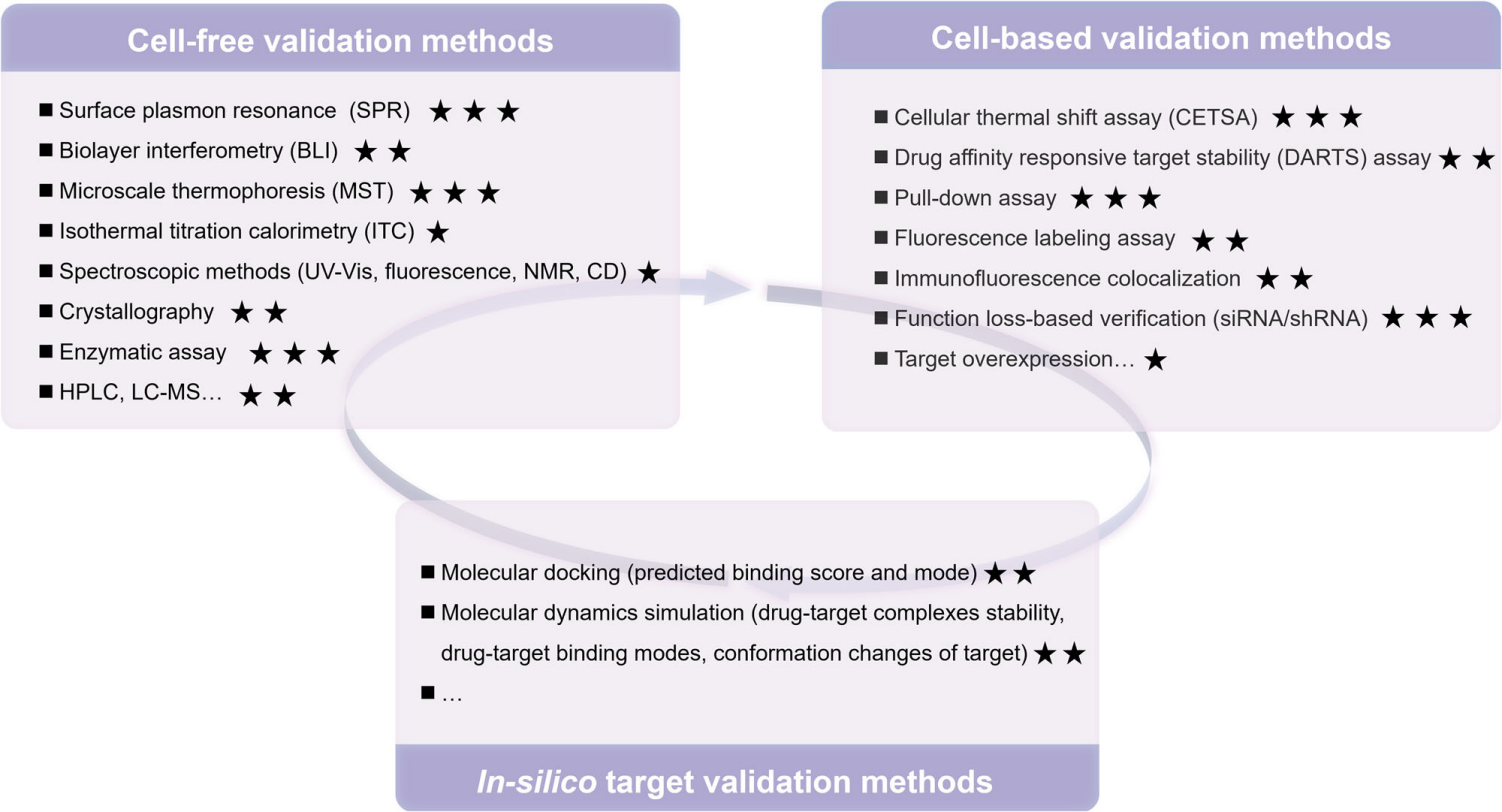
Summary and recommendation of validation methods for drug‐target interaction, including cell‐free validation methods, cell‐based validation methods, and in silico target validation methods. Recommended star ratings for different validation methods are based on a combination of accuracy, difficulty, and cost of target binding validation.

Typically, the target discovery of NPs using various approaches often leads to the identification of not just one target but multiple or numerous potential targets, and this is also the case with CEL. Although NPs have been accepted as molecules with multi‐target binding properties, the presence of experimental manipulation errors and false positive binding of proteins can lead to the identification of multi‐targets that may not always be accurate or may not necessarily represent the primary target responsible for producing a specific pharmacological effect. Hence, it is essential to employ a range of validation methods to furnish additional evidence of drug‐target interactions after target identification, thereby mitigating false positives and off‐targets. Essential criteria for target validation involve demonstrating the direct binding of NPs to the target protein and demonstrating that NPs exert a distinct pharmacological effect mediated by the target. This is imperative for uncovering authentic NP targets and accurately elucidating mechanisms of action. In this context, we have outlined commonly utilized methods for validating CEL targets in Figure 5 and Table 1 and categorized them into three groups: cell‐free validation, cell‐based validation, and in silico validation, with the expectation of providing effective guidance for drug‐target binding validation after target discovery of NPs. It is important to emphasize that a single validation approach may not suffice to establish conclusive evidence of drug‐target binding; hence, it is highly recommended to employ a combination of multiple validation methods for comprehensive validation.

### 4.1 Cell‐Free Validation Methods

Cell‐free validation refers to the measurement of ligand‐target interactions in an acellular environment (mainly recombinant proteins), including surface plasmon resonance (SPR), biolayer interferometry (BLI), microscale thermophoresis (MST), isothermal titration calorimetry (ITC), crystallography and enzymatic assay. SPR and BLI are commonly used methods to directly detect binding interactions between ligands and receptors immobilized on biosensor chips, which gives the ligand‐receptor binding affinity expressed by the equilibrium dissociation constant (*K*_D_) value [68]. These two methods are preferred for confirming the binding interaction between CEL and its targets and have been commonly employed in target discovery studies of CEL [17, 21, 28, 28, 45, 52, 54, 61]. MST is a cost‐effective and widely adaptable technique for the detection of ligand‐receptor affinity based on the microthermophoresis effect of biomolecules. It allows direct measurement of intermolecular interactions in solution without the need for any surface fixation [69]. ITC is currently recognized as the “gold standard for measuring ligand‐receptor interaction” and is based on the measurement of the heat released or absorbed by a sample cell containing receptors following ligand injection, providing rich thermodynamic information on the ligand‐receptor binding reaction, including binding energy, enthalpy change, entropy change, binding constant, and other thermodynamic parameters [70]. X‐ray crystallography can provide direct information on the three‐dimensional structure of protein‐ligand complexes at the atomic level, which is a powerful tool for indicating drug‐target binding and their interaction modes [68]. Xu et al. [54] elucidated the crystal structure of PRDX1 in a complex with CEL (resolution 1.76 Å), which provided strong evidence that CEL is a covalent inhibitor of PRDX1 with the binding site of Cys‐173. The enzymatic assay is the most direct way to determine the effect of drug binding on the activity and function of target proteins (activation or inhibition). For example, it is frequently employed to validate the binding between CEL and PRDXs family [17, 20, 28, 54]. Apart from the aforementioned techniques, additional methods for confirming the target engagement of CEL encompass HPLC, LC‐MS, UV–vis, nuclear magnetic resonance (NMR) spectroscopy, circular dichroism (CD) spectroscopy (Table 1).

### 4.2 Cell‐Based Validation Methods

Cell‐based validation refers to the direct or indirect measurement of target engagement in the physiological environment (intact cells, cell lysates, and tissues), including CETSA, DARTS, pull‐down, fluorescence labeling assay, immunofluorescence colocalization, and function loss‐based verification. CETSA is a label‐free biophysical assay for detecting ligand‐target binding based on the principle of ligand‐induced enhancement of thermal stabilization of target proteins, and it is highly recommended for the validation of NP‐target interaction [4]. For example, CEL was found to significantly increase the resistance of target proteins such as PRDXs (1,2,4,6) [17a,e], VDAC2 [17f], CAP1 [36], and P5CDH [52] to thermal denaturation, thus demonstrating direct binding of CEL to these proteins at the cellular level. DARTS, coupled with Western blot detection, is another label‐free biophysical assay for direct validation of targets, based on the principle that target proteins bound to NPs show greater resistance to enzymatic degradation compared to unbound proteins. However, a significant limitation of DARTS is that it can only be performed in cell or tissue lysates and cannot directly detect target binding in living cells [50, 57, 60, 71]. In a pull‐down assay for target validation, a designed probe of NPs, such as biotin or alkynyl‐conjugated NPs or immobilized NPs, is used to validate whether candidate proteins can be hooked directly from cells; or a competition strategy is applied to observe whether the early addition of free NPs can reduce the binding of the probe to the candidate protein by competing with the probe [17b]. Pull‐down assay is widely used to validate the binding of CEL to target proteins, such as CAND1 [17c], Shoc2 [28a], PRDX2 [28b], STAT3 [28c], and CAP1 [36]. Fluorescence labeling assay is a similar validation method to pull‐down, with the difference that a fluorescent group (such as TAMRA‐azide) is bound to the probe by click chemistry after incubation of the alkynyl probe with the cells, which in turn detects the fluorescence to measure the intensity of the target protein that is hooked up to the probe, rather than using antibody‐based Western blotting [23, 65]. Immunofluorescence colocalization involves the application of immunofluorescence methodologies to identify the NPs probe and target protein within the cell individually, intending to assess their spatial proximity to ascertain if there is an overlap. This analysis helps in establishing the authenticity of the protein as a target protein. Through the application of this approach, researchers have established that the CEL probe exhibited spatial overlap with HMGB1 [17b], PKM2 [17e], PRDXs [17a], CAND1 [17c], and Nedd4 [17d], thereby confirming the interaction of CEL with these proteins. However, both pull‐down and immunofluorescence colocalization require the synthesis of probes of NPs, thereby presenting a challenge to their widespread use. It is important to note that the binding of NPs to proteins does not necessarily modulate their function, therefore, functional experiments are also needed to confirm whether the identified proteins are specific targets for the biological function of NPs [15b]. Function loss‐based verification of target proteins is commonly achieved by target gene knockdown/knockout experiments using siRNA, shRNA, or CRISPR/cas9. Function enhancement‐based verification can be achieved by target overexpression using gene expression plasmids. The biological function of the candidate proteins related to the disease phenotypes can be revealed by observing the physiological and biochemical changes caused by gene silencing or overexpression. Moreover, by observing whether the function loss or overexpression of a specific protein can impact drug activity it can indirectly provide evidence that the protein is a drug target. For example, the essential role of IL1R1 in mediating the leptin‐sensitizing and anti‐obesity effects of CEL was validated using the knockout mouse model by Feng et al., underscoring the critical involvement of this target [49]. The knockdown of CAND1 in pathological cells could decrease the inhibitory effect of CEL on fibroblast‐myofibroblast transformation (FMT), providing evidence that the anti‐pulmonary fibrosis activity of CEL was dependent on CAND1 [17c]. In addition, co‐immunoprecipitation [44] is considered as available cellular target validation methods (Table 1).

### 4.3 In Silico Target Validation Methods

In silico target validation refers to the use of virtual computational techniques to characterize the interaction and binding modes of NPs with target proteins, where the results are predicted rather than real. Molecular docking and molecular dynamics simulation are two of the most commonly used validation methods (Table 1). Molecular docking is a common tool used to predict the interaction between a small molecule and a protein at the atomic level according to the “lock” and “key” principles [72]. Molecular dynamics is an upgraded computational technique that simulates the dynamic behavior of molecular systems as a function of time [73]. Molecular docking and molecular dynamics simulations can provide rich information on the binding affinity of NP‐protein, the stability of NP‐protein complexes, the behavior of NPs at target protein binding sites, and even conformation changes induced by NP binding, which provides strong support for the target validation, although these interactions are predictable. In addition, predictive information on amino acids with significant binding to NPs can also provide guidance for the implementation of subsequent amino acid mutations aimed at experimentally determining the specific binding site of the drug to the target. For instance, using molecular docking and molecular dynamics simulations, Ye et al. [28c] elucidated the intricate interactions between CEL and STAT3, and the computational predictions indicated a potential affinity of CEL towards Leu207 in the CCD domain and Gln635 and Val637 in the SH2 domain. Subsequent site mutation analysis (mutating these key amino acids to Ala) and pull‐down assays combined to strengthen the predictions, confirming Leu207, Gln635, and Val637 as the definitive binding sites for the CEL‐STAT3 interaction.

## 5 Network Analysis of CEL Targets Associated With its Therapeutic Effects and Side Effects

### 5.1 Network Analysis of Therapeutic Targets of CEL and Determination of Key Targets

**FIGURE 6:**
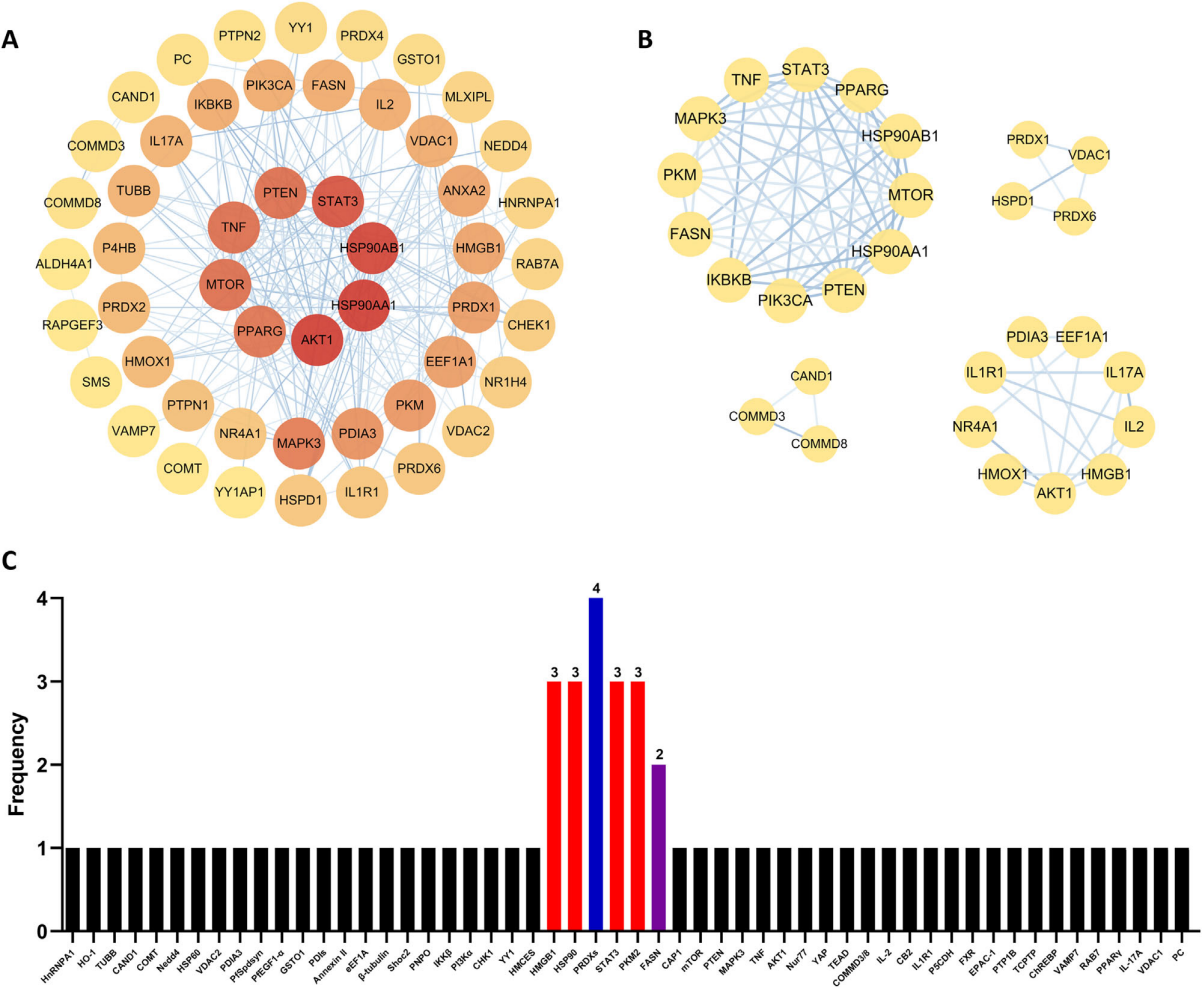
Network analysis and frequency analysis of the collected CEL targets. (A) Protein–protein interaction (PPI) network of CEL targets. The redder the node, the more important the targets in the network. (B) Clustery analysis of CEL target network using the MCODE plug‐in. (C) Frequency analysis of CEL targets in the publications.

To date, dozens of targets of CEL have been identified using different target discovery strategies, which significantly suggests CEL functions as a multi‐target therapeutic agent. However, the interrelations among these targets and their implications for disease treatment from a holistic perspective remain unclear. In addition, it is important to recognize that not all targets exert equal therapeutic effects. Therefore, clarifying the hub targets of CEL is essential to further advance CEL‐based drug discovery and development. In this section, we holistically analyzed the network interactions of the collected 59 CEL targets as listed in Table 1, which aims to gain insights into how CEL influences these targets to exert its therapeutic effects. As shown in Figure 6A, the protein‐protein interaction (PPI) network of collected CEL targets was established using the STRING database, comprising 51 nodes representing distinct targets and 222 edges denoting target‐target interactions. Each node's color corresponds to its degree of centrality within the network. Topological analysis of the network revealed an average node degree of 8.7, with hub nodes identified based on a criterion of greater than twice the average degree, including AKT1 (degree = 26), HSP90AA1 (degree = 26), HSP90AB1 (degree = 25), STAT3 (degree = 23), MTOR (degree = 19) PTEN (degree = 19). Furthermore, cluster analysis using the Cytoscape plug‐in MCODE was conducted to identify sub‐networks closely linked to the therapeutic effects of CEL (Figure 6B). The analysis demonstrated significant overlap between the highest‐scoring clusters identified by MCODE and the distribution of the eight hub nodes, reinforcing their pivotal roles in the target network of CEL. In addition, we analyzed the frequency of these targets in the publications related to the target discovery of CEL. It was observed that the majority of the targets were documented only once, with PRDXs, HMGB1, HSP90, and STAT3, PKM2 being the exceptions, having been reported three or more times in different sample types (Figure 6C). Notably, PRDXs were identified through a range of techniques, including ABPP chemical proteomics, protein microarrays, and multi‐omics analyses. HMGB1 was pinpointed as a direct target of CEL via chemical proteomics approaches in various sample types. HSP90 was identified as a CEL target through chemical proteomics and hypothesis‐driven confirmation methods. Similarly, STAT3 was recognized as a target through protein microarrays, network pharmacology, and hypothesis‐driven confirmation (Table 1). These targets have been identified in multiple laboratories using different methods, demonstrating their high confidence as key CEL targets. In summary, it is postulated that PRDXs, HMGB1, HSP90, STAT3, PKM2, AKT1, PTEN, and mTOR may serve as pivotal targets of CEL in mediating therapeutic effects across different diseases, as suggested by target network and frequency analyses.

Peroxiredoxins (PRDXs 1–6) are widely distributed oxidoreductase protein that serves as a significant enzymatic antioxidant defense mechanism in humans against reactive oxygen/nitrogen species. Apart from their established function in scavenging peroxides, PRDXs participate in modulating diverse cellular signaling pathways and contribute significantly to the pathogenesis of inflammatory diseases, cancer, and neurological disorders [74]. Consequently, they are viewed as a promising target for the treatment of a wide range of diseases. It was found that CEL directly binds to PRDX1, PRDX2, PRDX4, and PRDX6 through the active cysteine site, where CEL mainly covalently binds to the Cys83 and Cys173 sites of PRDX1 [17, 54], whereas the main covalent binding site with PRDX2 is Cys172 [17, 28]. Given that the Cys‐SH motif of PRDXs serves as the primary site for H_2_O_2_ oxidation, the covalent modification of CEL with these cysteine residues led to a reduction in the antioxidant activity of PRDXs. This resulted in an increase in reactive oxygen species (ROS) levels in cancer cells, leading to cell death and ultimately exhibiting anti‐cancer effects. In addition, the inhibition of PRDX activity by CEL promoted ROS production and induced iron death in activated hepatic stellate cells (HSCs), thereby exerting an anti‐hepatic fibrosis effect [17a].

High mobility group protein B1 (HMGB1) is a highly abundant and extensively studied HMG protein that serves various crucial functions within the cell, such as acting as a DNA chaperone, safeguarding chromosomes, maintaining autophagy, and protecting against apoptosis. Additionally, it plays a pivotal role as a prototypical damage‐associated molecular pattern molecule (DAMP) outside the cell. Consequently, targeting the expression, release, and activity of HMGB1 has emerged as a promising therapeutic approach for a diverse array of human ailments, including inflammation, infectious diseases, immune disorders, neurodegenerative diseases, metabolic disorders, and cancers [75]. CEL does not affect the expression and secretion of HMGB1 but can form covalent adducts with Cys residues (Cys 23/45/106) of the A box and B box of HMGB1, thereby blocking the binding of HMGB1 to the inflammatory receptor and inhibiting its pro‐inflammatory activity, which exerts a significant therapeutic effect in neurological and inflammatory diseases [17b,e].

HSP90α (HSP90AA1) and HSP90β (HSP90AB1) are key members of the heat shock protein (HSP) family, involved in various physiological processes, with their malfunction closely associated with the progression of numerous diseases such as cancer and neurodegenerative diseases [76]. Previous studies have linked the antitumor properties of CEL to its actions to inhibit cancer cell growth, induce apoptosis, and reduce inflammatory responses, and these actions are closely associated with the modulation of HSP90 activity [20, 56]. Furthermore, HSP90 is involved in the regulation of inflammatory responses and cellular stress by CEL, which is particularly relevant to therapeutic interventions for hepatitis C virus (HCV) infection [57]. Signal transducer and activator of transcription 3 (STAT3), a pivotal member of the STAT transcription factor family, is ubiquitously expressed in diverse cell types and tissues, playing a crucial role in the JAK/STAT signaling cascade, which is significantly activated in various pathological conditions like cancer and inflammation, thereby exacerbating disease development. Therapeutics targeting STAT proteins hold a prominent position in contemporary drug discovery efforts [77]. It was found that Leu‐207 in the CCD domain and Gln‐635/Val‐637 in the SH2 domain play important roles in the binding of CEL to STAT3 and that CEL exerted cardioprotective and anti‐cancer effects by directly binding to and inhibiting the phosphorylation and nuclear translocation of STAT3 [28, 59].

Pyruvate kinase M2 (PKM2) is a crucial enzyme involved in glycolysis, significantly influencing cellular metabolism and proliferation. Recent studies have highlighted the role of PKM2 in the progression of various diseases, particularly in cancer, inflammatory disorders, and cardiovascular conditions [78]. This underscores the potential of PKM2 as a therapeutic target, with the development of small molecule inhibitors or agonists aimed at PKM2 offering promising avenues for novel treatment options for these diseases. Research has demonstrated that CEL is bound to the cysteine residue Cys424 of PKM2, resulting in the inhibition of enzymatic activity and subsequently suppressing PKM2‐dependent aerobic glycolysis, commonly referred to as the Warburg effect. This inhibition has been shown to protect mice from sepsis inflammation [17e]. The Warburg effect, characterized by enhanced aerobic glycolysis, is also a significant contributor to metabolic dysfunction and inflammatory stress in nonalcoholic fatty liver disease (NAFLD). CEL has been observed to covalently bind to the Cys31 residue of PKM2, leading to alterations in the spatial conformation of the enzyme and a consequent reduction in its activity. Through its interaction with PKM2, CEL has been found to significantly diminish glycolytic signaling, thereby mitigating lipid accumulation, inflammation, and fibrosis within the liver [26]. PKM2 is integral to the metabolic reprogramming of cancer cells, with its expression and activity closely linked to tumor proliferation, metastasis, and resistance to therapeutic agents. Consequently, the modulation of PKM2 activity is regarded as a potential strategy for cancer treatment [79]. However, there is currently a lack of evidence regarding the antitumor effects of CEL through the regulation of PKM2, and further investigation into the implications of the interaction between CEL and PKM2 in tumor progression is warranted.

PI3K/AKT signaling pathway is commonly accepted as a critical pathway implicated in the pathogenesis of cancer, inflammatory diseases, neurodegenerative diseases, autoimmune disorders, and metabolic conditions [80]. Serine/threonine‐protein kinase (AKT1) is an important component of the PI3K/AKT pathway, while PTEN is a negative regulator of the pathway. Serine/threonine‐protein kinase mTOR is a key downstream signaling molecule of PI3K/AKT, which is closely related to tumor progression. A network pharmacology analysis of CEL has indicated potential interactions with these three targets [39, 41]. Furthermore, the DBPP strategy has also identified PI3Kα as a target of CEL's action [32]. These results suggest a broad spectrum of inhibitory effects of CEL on the PI3K/AKT signaling pathway, thereby yielding beneficial treatments for a variety of diseases.

### 5.2 Bioinformatics Analysis of Therapeutic Targets of CEL

**FIGURE 7:**
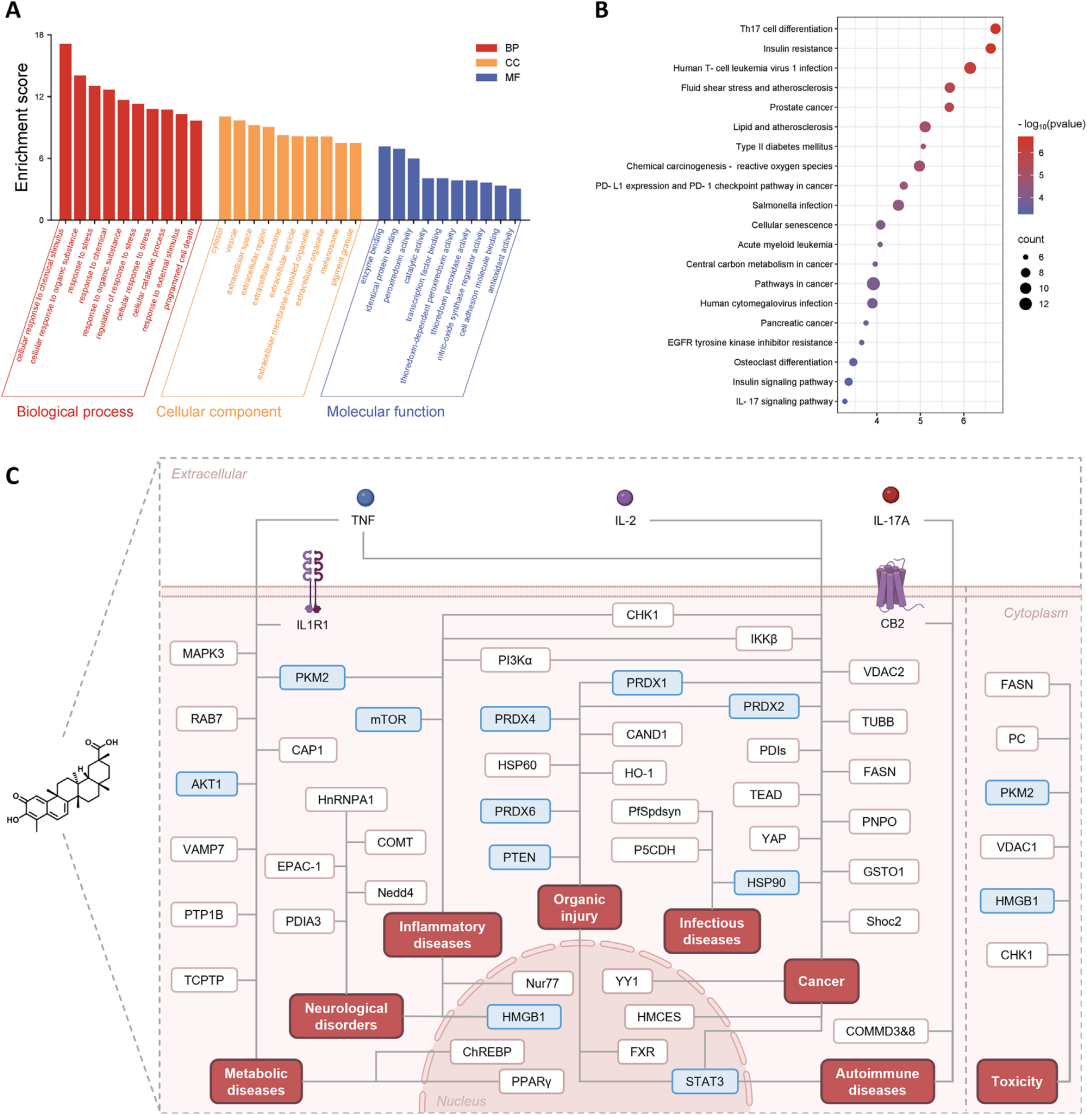
Analysis of collected therapeutic or toxicological targets of CEL. (A) GO enrichment analysis of CEL targets. (B) Top 20 enriched pathways from KEGG pathway enrichment analysis. (C) An established “target‐disease” network of CEL. The connecting lines represent the direct relationship between the target and pharmacological or toxicological actions. Key targets are highlighted in blue, including PRDXs, HMGB1, HSP90, STAT3, PKM2, AKT1, PTEN, and mTOR.

To reveal the overall role of the collected targets, gene ontology (GO) enrichment analysis and Kyoto Encyclopedia of Genes and Genomes (KEGG) pathway enrichment analysis were carried out following target network analysis. GO enrichment results revealed a significant enrichment of 557 GO entries, encompassing 479 biological processes (BP), 43 molecular functions (MF), and 35 cellular components (CC). The top ten enriched GO terms were visualized using bar graphs (Figure 7A). Analysis results suggested that CEL may exert its pharmacological effects by modulating multiple biological processes, including cellular response to chemical stimuli, cellular response to organic substance, response to stress, response to chemicals, cellular catabolic process, and programmed cell death. The molecular functions of these CEL targets predominantly involve enzyme binding, identical protein binding, PRDX activity, catalytic activity, and transcription factor binding. In addition, in KEGG pathway enrichment analysis, 68 significantly enriched signaling pathways were identified and the top 20 enriched KEGG pathway terms are listed in a bubble plot (Figure 7B), such as Th17 cell differentiation, insulin resistance, human T cell leukemia virus 1 infection, chemical carcinogenesis‐reactive oxygen species, pathways in cancer, osteoclast differentiation, IL‐17A signaling pathway. These results indicate that the potential pharmacological effects of CEL against various disease conditions are closely associated with the regulation of these signaling pathways, including cancer, inflammation, autoimmune diseases, metabolic diseases, infectious diseases, and neurodegenerative diseases. Based on the above results, we established a comprehensive “target‐disease” network of CEL. In this network, the association between reported targets and relevant diseases was established, allowing researchers to look for “clues” as to how CEL might treat a particular disease. It is highlighted that the broad and significant therapeutic effects of CEL on different diseases involve the complex modulation of multiple targets and multiple signaling pathways rather than only acting on a single target or a single signaling pathway (Figure 7C).

### 5.3 Toxicological Target Analysis of CEL

Currently, only HMGB1, VDAC1, PC, PKM2, FASN, and CHK1 have been found to be associated with toxicological effects of CEL, including reproductive toxicity and nephrotoxicity, through several target discovery strategies such as ABPP and DBPP (Figure 7C). Voltage‐dependent anion‐selective channel protein 1 (VDAC1), a multifunctional protein situated on the outer mitochondrial membrane, plays a critical role in preserving the structural and functional integrity of mitochondria. It is integral to various processes, including energy production, regulation of mitochondrial oxidative stress, calcium transport, metabolic processes, apoptosis, and mitophagy [81]. Research indicates that CEL can covalently bind to the cysteine residues of VDAC1, leading to mitochondrial dysfunction [66]. Pyruvate carboxylase (PC) is essential for sugar isomerization and the tricarboxylic acid cycle, while fatty acid synthetase (FASN) is involved in fatty acid β‐oxidation, and PKM2 serves as a pivotal enzyme in glycolysis. Collectively, these enzymes are crucial for regulating cellular energy supply and maintaining metabolic homeostasis, thereby supporting normal physiological functions. However, CEL's targeting of these key metabolic enzymes at non‐disease sites disrupts energy supply and metabolic synthesis, resulting in significant damage to vital organs, particularly the kidneys [66]. HMGB1 is a significant nuclear protein that participates in various biological processes, including the regulation of gene expression, DNA repair, and cell proliferation [75b]. By targeting HMGB1 and disrupting its regulatory function on p21 transcription, CEL has been shown to induce ovarian insufficiency and granulosa cell apoptosis, thereby contributing to reproductive toxicity [65]. Checkpoint Kinase 1 (CHK1) is a member of the serine/threonine kinase family and serves as a crucial regulatory protein in the cell cycle, primarily involved in cellular responses to DNA damage and replication stress [82]. It was found that high concentrations of CEL result in the down‐regulation of CHK1 protein levels, which subsequently induced a notable G2/M phase arrest, ultimately resulting in cytotoxicity [32]. However, CEL has been shown not to inhibit the kinase activity of CHK1, and how CEL interacts with CHK1 still needs to be revealed in the future.

It is noteworthy that HMGB1, PKM2, and FASN, while demonstrated to be associated with the toxicity of CEL, have also been recognized as targets through which CEL can exert a range of pharmacological effects, including neuroprotection, antisepsis, antitumor activity, and anti‐NAFLD properties (Table 1). CEL may provide beneficial therapeutic outcomes by interacting with targets located at disease sites; however, its interactions with targets at non‐disease sites may result in adverse side effects, indicating a dual role of the same target in varying pathological contexts. A more comprehensive discussion of these targets is warranted in future research to elucidate the mechanisms by which CEL can produce either therapeutic or toxic effects. Furthermore, it is essential to devise strategies aimed at mitigating the toxicity of CEL while maintaining its therapeutic efficacy. One potential approach could involve the coadministration of CEL with other pharmacological agents to reduce its interaction with toxic targets, thereby enhancing both safety and efficacy. Additionally, strategies that promote the accumulation of CEL at disease sites while minimizing its distribution in non‐targeted organs could significantly improve its biosafety profile. Achieving such an aim requires the help of novel nano‐delivery systems. Moreover, the target selectivity of CEL can be further improved by optimizing the structure of CEL, which aims to enhance the interaction with therapeutic targets while preventing binding to toxic ones. Nonetheless, any strategy must be grounded in a thorough understanding of the toxicity target profile of CEL, underscoring the imperative for pharmacological researchers to intensify their focus on the targeting studies of CEL in future investigations.

## 6 Clinical Trial Information of CEL

Despite the promising therapeutic potential of CEL for a variety of diseases, as demonstrated in preclinical studies, several challenges persist that hinder their clinical application. These challenges include poor water solubility, limited bioavailability, limited efficacy, incomplete understanding of its mechanism of action, and adverse effects associated with CEL use. Currently, clinical trials involving CEL are still in the early stages, with limited independent studies, and its safety and efficacy in humans have not been fully established. Despite these limitations, existing research provides preliminary evidence supporting its potential for clinical use. For instance, a study involving 276 patients demonstrated that the combination of CEL and oral nifedipine led to a significant reduction in hypertension among patients with pregnancy‐induced preeclampsia. Administration of CEL did not result in an increased incidence of severe maternal or neonatal adverse reactions, which supported CEL might serve as an adjuvant to oral nifedipine against preeclampsia [83]. Preclinical investigations have demonstrated that CEL, functioning as a leptin sensitizer, can significantly promote weight loss in obese murine models [6]. To further assess its potential for clinical application, ERX‐1000 (CEL or its derivatives), a first‐in‐class leptin sensitizer, is in development for the treatment of obesity and related diseases. Initial clinical findings have indicated a dose‐dependent reduction in weight over 4 weeks, thereby validating the efficacy of ERX‐1000 in individuals with obesity (https://www.erxpharmaceuticals.com/research‐development/erx‐1000‐for‐obesity/). In addition, a phase II/III clinical trial (IRCT20220424054643N2) is currently underway to assess the safety and efficacy of a CEL solution in patients with fractures. Since the 1970s, tripterygium glycoside tablets (TGT), containing CEL as a bioactive component, have been employed for arthritis management. Clinical studies on TGT have shown notable therapeutic effects in treating renal diseases and autoimmune disorders, with good safety profiles when used in combination with other treatments.

Currently, clinical investigations concerning CEL remain markedly inadequate. Considering that how CEL exerts its therapeutic effects on diseases has not yet been fully elucidated, an in‐depth analysis of CEL's targets and pharmacological mechanisms remains an important basis for improving its potential for clinical application. Investigating the effects of CEL in combination with other pharmacological agents and assessing the synergistic outcomes of combination therapies represents an effective strategy for advancing clinical applications of CEL. Additionally, the exploration of novel dosage forms, such as nanomedicines and extended‐release formulations, as well as novel routes of CEL administration, may significantly enhance drug bioavailability and patient adherence, thereby broadening the scope of CEL's clinical applications. It is imperative to strengthen the design and execution of large‐scale, randomized, controlled clinical trials to substantiate the efficacy and safety of CEL, as this is a crucial step toward the successful translation of CEL into clinical therapeutics. Furthermore, the establishment of a systematic platform for monitoring adverse effects is vital for ensuring the safe application of CEL. In summary, the progression of CEL into clinical practice necessitates interdisciplinary collaboration among pharmacology, clinical medicine, and materials science. It is anticipated that as further clinical trials and long‐term follow‐up data come to light, the clinical applications of CEL will receive stronger support, thus facilitating a comprehensive evaluation of its therapeutic efficacy and safety.

## 7 Drug Combination With CEL Based on Target Discovery

**FIGURE 8:**
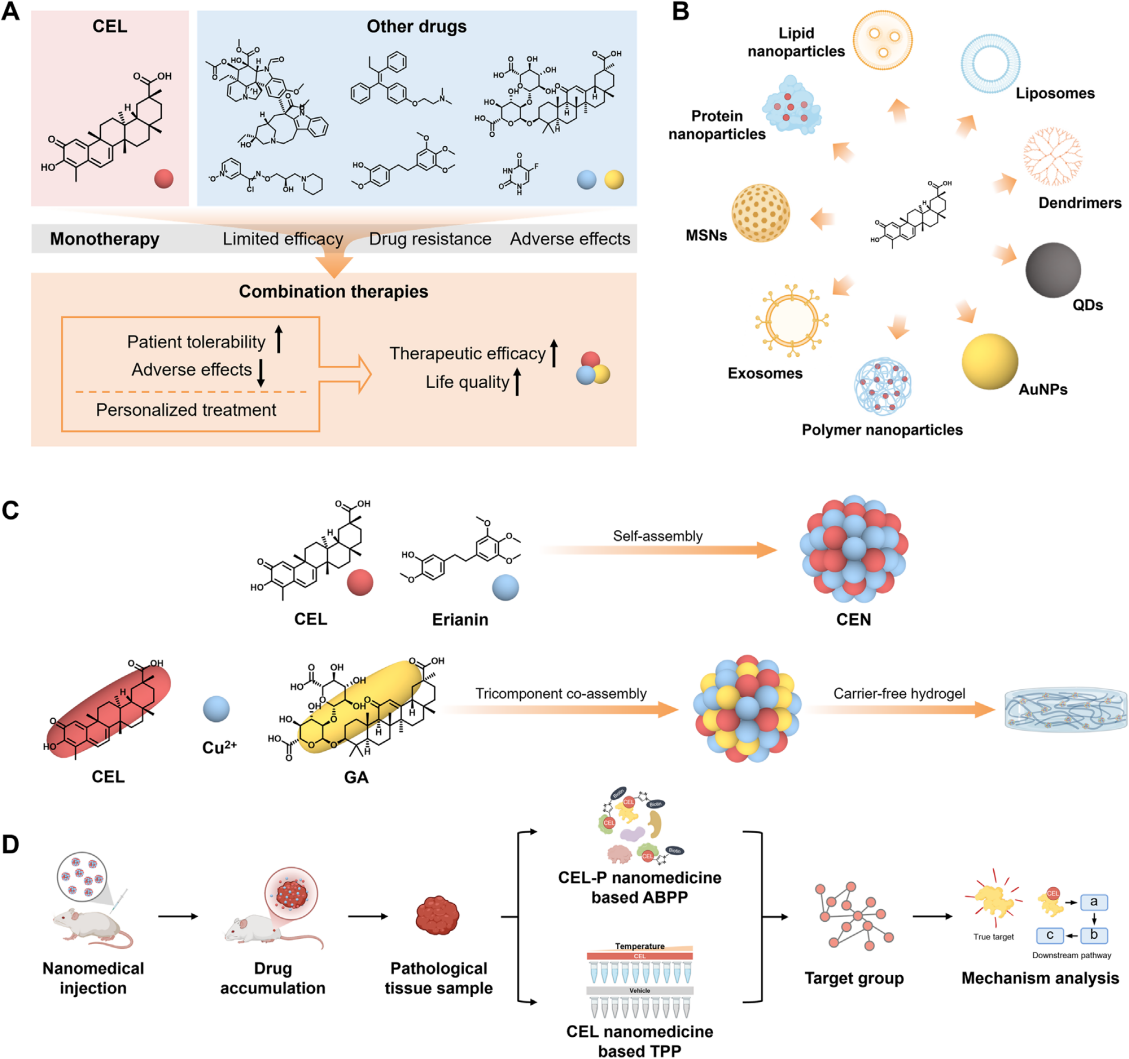
Drug combination and drug delivery of CEL based on target discovery. (A) Combination therapies with CEL. (B) Reported nanocarrier systems for CEL delivery. (C) Carrier‐free self‐assembled CEL nanomedicine for CEL delivery. (D) Target discovery promotes answers to the mechanism of action of CEL nanomedicines.

Drug combination refers to the strategy of the simultaneous administration of two or more pharmacological agents to treat a specific disease. This approach can enhance therapeutic outcomes by utilizing drugs with distinct mechanisms of action, particularly in the treatment of cancer, infectious diseases, and chronic conditions. Monotherapy often leads to the development of drug resistance; however, the use of combination therapies can mitigate this issue by targeting multiple pathways. Furthermore, the adverse effects of individual drugs can be alleviated through combination strategies, such as employing one drug to counteract the side effects of another, thereby improving patient tolerability and overall quality of life. Additionally, drug combinations allow for the personalization of treatment regimens based on individual patient conditions and biomarkers, which can enhance therapeutic efficacy and minimize adverse effects. Consequently, investigating the potential of combining CEL with other pharmacological agents may offer a viable pathway for the effective clinical application of CEL in disease management (Figure 8A).

In recent years, some cases have been reported in which CEL has been utilized in combination with other pharmacological agents to enhance therapeutic efficacy, mitigate adverse effects, and diminish the development of drug resistance. For example, CEL has been used in combination with clinical antitumor drugs such as vincristine, 5‐FU, and tamoxifen to enhance the sensitivity of these drugs and reduce drug resistance, thus improving the antitumor effect [84]. Additionally, CEL has been combined with arimoclomol for the management of neurodegenerative disorders [85]. Despite some promising outcomes, the combination of CEL with other drugs is often a low‐throughput serendipitous endeavor, and the mechanism of action of drug combinations remains obscure. Recent advancements in drug target discovery methodologies have introduced novel concepts and tools for investigating drug combinations, which are anticipated to facilitate the formulation of more effective therapeutic strategies in the future. On the one hand, the optimization of drug combinations cannot be separated from the elucidation of CEL target profiles by various target discovery techniques. A thorough understanding of CEL's target information can assist researchers in selecting complementary drugs with distinct mechanisms of action, thereby avoiding the repetitive selection of agents targeting the same pathways and maximizing the potential for synergistic effects. For example, mTOR is one of the key targets for CEL to exert pharmacological effects. By combining the mTOR inhibitor CEL with the TGF‐β receptor I (TGFβRI) inhibitor (LY2157299), the anti‐glioma effect was significantly improved [86]. On the other hand, target discovery serves as a valuable approach for elucidating the mechanisms underlying the action of drug combinations. For instance, the synergistic effects of drug combinations on phenotypic outcomes and the regulation of signaling pathways can be elucidated through the detection of multi‐omics alterations induced by the drug combination. Employing proteome‐wide label‐free approaches such as TPP, researchers can delineate the target profiles of drug combinations that exhibit synergistic effects. By comparing these profiles with those of individual drugs, it becomes possible to predict the key targets responsible for the synergistic and toxicity‐reducing effects of the combinations. Furthermore, network pharmacology facilitates the analysis of the intricate relationships between drugs and their targets, enabling the identification of potential drug combinations and their mechanisms of action, thereby advancing the research and development of combination therapies. For example, Ning et al. utilized network pharmacology to identify MAPK1, AKT1, PIK3CB, EGFR, and VEGFA as effective targets for the anti‐colorectal cancer effects of CEL in combination with vincristine [84a]. Kim et al. demonstrated that the combination of CEL with β‐lactam antibiotics significantly increased the susceptibility of resistant bacterial strains to antibiotics. Mechanistically, whole‐genome sequencing of mutant strains revealed that CEL disrupted cell wall cross‐linking in bacteria by modulating c‐di‐AMP level, thereby functioning as a potentiator of antibiotics [87]. In summary, the strategy of target identification holds considerable promise to promote combination therapies, as it can be employed to identify and validate targets and optimize drug combinations, ultimately enhancing therapeutic efficacy and improving the quality of life for patients.

The design of rational drug combinations is critical for enhancing the effectiveness of combination therapies. In recent years, AI has demonstrated significant potential in various aspects of drug discovery, including target identification and drug repurposing. Ouyang et al. have recently introduced an innovative framework of target omics‐based intelligent drug pair discovery, offering a novel and effective methodology for drug combination design. This framework integrates large‐scale bioinformatics analysis with neural network AI to optimize drug pair selection. Utilizing this platform, the researchers have identified a favorable combination of mitoxantrone and gambogic acid for synergistic anti‐breast cancer effects through the modulation of cellular pyroptosis [88]. Building upon these findings, we propose a strategy that may be applicable to the design of drug combinations based on CEL. Briefly, this approach involves the identification of biomarkers and targets associated with specific diseases through the analysis of large‐scale omics data derived from clinical samples. This analysis leads to the generation of a characteristic target group comprising multiple high‐risk biomarkers linked to a particular phenotype. Subsequently, a library of potential drugs that exert interventional effects on characteristic target groups is created, either through target‐based high‐throughput drug screening or by utilizing publicly available drug‐target interaction databases, such as the Comparative Toxicogenomics Database (CTD). Further, various drug target discovery methods are utilized to thoroughly clarify the target information of CEL and the potential drug library. Following this, the interrelationships among genes, proteins, drug targets, and disease phenotypes are analyzed by introducing suitable AI models to realize the prediction of the combination effects of CEL and individual drugs and, ultimately, the discovery of potent drug combinations. By integrating diverse disciplines, including bioinformatics, big data, AI, and pharmacology, this strategy has the potential to significantly enhance the clinical application of CEL‐based drug combinations. Furthermore, this approach also supports the development of personalized combination drug regimens, thereby advancing the possibilities for drug combination design within the framework of precision medicine.

## 8 Drug Delivery of CEL Based on Target Discovery

Despite the promising therapeutic potential of CEL in various diseases, its clinical application is still hindered by challenges such as low water solubility, poor permeability, and limited oral bioavailability. Moreover, the limited targeting and off‐target side effects (e.g., cardiotoxicity, hepatotoxicity, and neurotoxicity) of CEL resulting from the large number of reported and undiscovered CEL targets and their wide distribution in various organs and tissues should not be overlooked [89]. The advent of nanotechnology‐based delivery strategies offers a promising approach to enhance the oral bioavailability, therapeutic efficacy, and tissue‐specific targeting of CEL [89, 90]. With the continuous elucidation of the pharmacological effects of CEL and the increasing attractiveness of CEL in the treatment of complex diseases, drug delivery studies of CLE have gradually become a hot spot in recent years. Various nanocarrier systems have been developed for CEL delivery, including liposomes, exosomes, polymer nanoparticles, lipid nanoparticles, protein nanoparticles, dendrimers, gold nanoparticles (AuNPs), mesoporous silica nanoparticles (MSNs), and quantum dots (QDs) (Figure 8B). The continuous advancement of these nano‐delivery platforms has been instrumental in addressing the limitations of CEL's clinical use and facilitating its transition into viable clinical therapeutics. The research results achieved in the field of drug delivery for CEL have been summarized and discussed in detail in several reviews [89, 91]. Therefore, herein, we focus on the design of drug delivery systems for CEL based on reported target information and discuss the potential application of target identification techniques in the rational design and mechanism of action elucidation of CEL nano‐drugs.

### 8.1 Target Information Guides the Rational Design of Nano‐Delivery Systems for CEL

A comprehensive comprehension of the pharmacological effects and underlying mechanisms of drugs is essential for the rational development of drug delivery systems. With the help of emerging target discovery strategies, the target‐centered mechanisms of CEL for the treatment of complex diseases have been elucidated in depth, which provides effective guidance for researchers to design reasonable and effective nanocarriers encapsulating CEL based on its specific mechanism of action for a particular disease. For instance, by leveraging CEL's ability to inhibit NF‐κB signaling to impede tumor metastasis and growth, Zhao et al. developed an αvβ3 receptor‐targeted TET‐CSOSA/CEL micelle that effectively blocked the NF‐κB signaling pathway, leading to a substantial reduction in lung metastasis and situ tumor growth in breast cancer. This approach presents a promising strategy for concurrently treating lung metastasis and primary tumors [92]. In a separate investigation, VDAC2 was identified to be a critical anticancer target of CEL. By targeting VDAC2, CEL induced iron prolapse and cancer cell apoptosis, thereby manifesting its anti‐hepatocellular carcinoma properties. Leveraging this insight, researchers employed alkyl glucoside‐modified liposomes (AGCL) to encapsulate CEL, resulting in AGCL exhibiting notable hepatocellular carcinoma targeting capabilities and enhanced anti‐tumor efficacy while minimizing adverse effects [17f]. By RNA sequencing analysis, CEL was found to inhibit PI3K/AKT/mTOR signal and promote autophagy in myofibroblasts. Based on this finding, an injectable thermoresponsive‐hydrogel was constructed for the in situ release of CEL and showed significant prevention of corneal scarring and corneal stromal fibrosis in lamellar keratoplasty [93].

As mentioned earlier, drug combination is an effective strategy to promote the translation of CEL to clinical applications, but how to achieve efficient synergistic delivery of co‐administered drugs remains a great challenge. In recent years, there has been considerable interest in the utilization of nano‐delivery platforms for the design and development of these drug combinations. By loading multiple drugs, enhancing drug solubility and bioavailability, improving targeting to specific cells or tissues, and regulating drug release, nano‐delivery systems can substantially augment the synergistic effects of drug combinations while minimizing adverse reactions. The decrypted CEL target information can provide meaningful references for the design and construction of CEL co‐delivery systems with other clinical drugs or target inhibitors. Drawing from the insights regarding the therapeutic target information of CEL, it is possible to select pharmacological agents with different mechanisms of action for co‐administration, thereby facilitating the design and optimization of combination therapies. Following this, CEL and the selected co‐administered drugs are encapsulated within the same nanocarrier, enabling simultaneous intervention on multiple targets and signaling pathways for the treatment of complex diseases. By elucidating the therapeutic target at the site of disease and the off‐target effects on non‐target organs or cells, nanocarriers with specific targeting characteristics are engineered for precise delivery. This approach aims to enhance the efficacy of co‐delivery drugs on the intended therapeutic target while minimizing toxicity to non‐target cells or tissues.

By taking advantage of CEL's ability to inhibit tumor growth and proliferation and resist drug resistance through the modulation of NF‐κB signaling, researchers have coupled docetaxel (DTX) and CEL with lactoferrin to construct a lactoferrin‐dual drug nanoconjugate, which not only significantly improved the solubility, loading capacity, and anti‐breast cancer effect of DTX and CEL, but also effectively overcame the drug resistance challenge of DTX [94]. In our previous study, a hyaluronic acid‐based dissolvable microneedle (MN) was constructed for the co‐delivery of CEL and PUMA‐expressing plasmid, which realized the combination of CEL and gene therapy in the treatment of RA. The designed microneedle effectively released the PUMA‐expressing plasmid, leading to the upregulation of PUMA expression, which subsequently induced apoptosis in synovial cells. Concurrently, it released CEL in the microacidic environment of the joints, which mitigated inflammation through the modulation of the NF‐κB pathway. This dual‐action approach resulted in a synergistic reduction of joint inflammation, as well as the preservation of cartilage and bone integrity [95]. Nur77 has been identified as the key target for CEL to exert anti‐tumor effects. In a study, Liu et al. [96] designed a smart drug delivery system (PPP/Nur77/CEL), which consisted of mPEG‐PCL‐PEI polymers loaded with the Nur77 gene and CEL. The system was characterized by the fact that CEL, in addition to acting as an anticancer drug, promoted the translocation of Nur77 expressed by the loaded Nur77 gene to the mitochondria and converted Bcl‐2 from an anti‐apoptotic protein to a pro‐apoptotic protein, thereby increasing the sensitivity of Bcl‐2 overexpressing cancer cells to the drug and achieving synergistic enhancement of both the chemotherapy and gene therapy. In another study utilizing the properties of CEL as an mTOR inhibitor, Zhu et al. [86] designed a biomimetic blood‐brain barrier‐penetrating albumin nanosystem for co‐delivery of a TGF‐β receptor inhibitor (LY2157299) and CEL. In this system, CEL could inhibit the STAT6 pathway and repolarize tumor‐associated macrophages from the M2 phenotype to the M1 phenotype, thus alleviating tumor immunosuppression, and exerting a synergistic and potentiating effect with LY2157299, which showed a good therapeutic effect on glioma. In addition, several other studies have utilized the properties of CEL to regulate PI3K/Akt signaling to design CEL‐based nanoporous membranes [97] or CEL‐CSO/Taxol nanoparticles [98], and these platforms have demonstrated significant potential for the treatment of eye diseases or drug‐resistant breast cancer. Collectively, by referring to the information on the identified targets of CEL, it can enable researchers to design and construct nano‐delivery systems more rationally by utilizing the diverse pharmacological properties of CEL, thereby advancing clinical drug development rooted in CEL. Furthermore, target information of drugs is instrumental in the selection of appropriate materials aimed at enhancing the drug's stability in vivo, thereby preventing degradation or inactivation before reaching the intended targets. The distribution and expression levels of the target can significantly affect the pharmacokinetic characteristics of a drug. Consequently, the optimization of the particle size, surface charge, and morphology of the nano‐delivery system can be optimized by referring to the target information to improve the absorption and distribution of the drug. These advantages underscore the critical importance of understanding target information in the design of nano‐delivery systems.

### 8.2 Target Information Drives the Development of Carrier‐Free Self‐Assembled CEL Nanomedicine

Although nano‐delivery systems have achieved remarkable results in addressing the medication drawbacks of NPs, traditional nano‐delivery systems predominantly utilize synthetic carriers (such as liposomes and polymers), which may pose additional challenges related to biocompatibility and biodegradability. Furthermore, these systems frequently exhibit limitations, including low drug loading capacity, which complicates their clinical application. To overcome these limitations, the emerging discovery of the self‐assembly phenomenon of NPs in recent years has brought new ideas for the development of NP drugs. Bioactive NPs not only demonstrate unique bioactivities but also possess the ability to self‐assemble through non‐covalent molecular interactions, resulting in the formation of various supramolecular bioactive materials, including nanoparticles, micelles, and hydrogels [99]. In contrast to carrier‐based delivery systems, these self‐assembled materials facilitate direct drug delivery, exhibit high drug loading capacities, and are straightforward to prepare, thereby reducing production costs. Furthermore, these supramolecular bioactive materials significantly enhance the solubility of NPs, improve biocompatibility, enable penetration of biological barriers, allow for controlled and responsive release, promote synergistic effects, and mitigate toxicity. In summary, carrier‐free self‐assembled nanomaterials represent a novel nano‐delivery system that offers a promising strategy to address the limitations associated with NPs, demonstrating substantial potential for clinical application and translation.

A variety of structural types of NPs have been found to form nanomedicines by self‐assembly, including polyphenols, quinones, saponins, alkaloids, terpenoids, and sterols. The self‐assembly of these NPs is facilitated by various supramolecular interactions, which include electrostatic interactions, hydrogen bonding, hydrophobic interactions, van der Waals forces, and metal‐ligand interactions [99a]. As a significant bioactive terpenoid, CEL has recently been identified for its capacity to self‐assemble into nanodrugs (Figure 8C). Utilizing the interaction between CEL and erianin in an aqueous environment, Tian et al. developed a carrier‐free celastrol‐erianin nanomedicine (CEN) by employing a nanoprecipitation technique [100]. Investigations into the self‐assembly mechanism revealed that the interaction between CEL and erianin was primarily driven by π–π stacking interactions and intermolecular hydrophobic forces, rather than hydrogen bonding interactions. The absence of additional carriers resulted in CEN demonstrating exceptionally high drug loading capabilities, with encapsulation and drug loading efficiencies of 96.45% and 63.15% for CEL, respectively. Upon entering the pathological environment, CEN was capable of releasing CEL and erianin. The released CEL triggered significant apoptosis in tumor cells, whereas erianin inhibited tumor proliferation and mitigated tumor invasion and metastasis. This synergistic effect of CEN led to a more pronounced inhibition of breast cancer progression compared to free drugs, while also reducing associated toxic side effects [100]. In addition, the researchers developed a metal‐coordinated carrier‐free CEL hydrogel, which comprises CEL, copper ions (Cu^2+^), and glycyrrhizic acid (GA). Mechanically, GA, Cu^2+,^ and CEL were first co‐assembled into tightly packed composite units in an end‐to‐end manner via coordination bond and hydrogen bond and then stacked to form spherical particles under the action of intermolecular forces, which eventually appeared as carrier‐free co‐assembled hydrogels. The resulting CEL hydrogels demonstrated remarkable material properties, including stability, shear‐thinning behavior, self‐healing capability, and injectability. This ternary carrier‐free small molecule hydrogel facilitated a multifunctional approach that integrates chemotherapy, chemo‐dynamic therapy, tumor microenvironment (TME) modulation, and immunomodulation, thereby exhibiting significant therapeutic efficacy against both primary and metastatic tumors, along with promising clinical translational potential [101].

The advancement of nanomedicines is not merely contingent upon serendipitous findings; rather, it necessitates a thorough investigation into the principles and factors influencing the self‐assembly of CEL in conjunction with other NPs. This includes elucidating the relationships between the structural characteristics of NPs and their assembly modes to predict the outcomes of molecular self‐assembly, which is essential for lowering costs and providing a theoretical foundation for a broad spectrum of future research endeavors. Furthermore, emerging methodologies for target discovery must be employed to delineate the therapeutic and toxicological target profiles of NPs comprehensively, thereby offering insights into the co‐assembly of multiple components. Based on the identified target data, the co‐assembly ability of CEL with other NPs or clinical drug molecules can be utilized to design synergistic therapeutic approaches for specific diseases. By designing and constructing binary, ternary, or even multi‐component self‐assembling nanomedicine, “multi‐component, multi‐target, and multi‐pathway” treatment strategies can be realized. Additionally, further investigation into the molecular mechanisms of nanomedicines is crucial for understanding their therapeutic effects. Concurrently, it is essential to encourage further research on the stability, biocompatibility, and safety of self‐assembled nanomedicines. Such investigations are crucial for advancing the clinical application of CEL nanomedicines.

### 8.3 Target Discovery Methodologies Promote Answers to the Mechanism of Action of CEL Nanomedicines

Investigating the in vivo fate of nano drugs and understanding how released drugs exert their pharmacodynamic effects in vivo poses a significant challenge in the realm of nanomedicine research. Presently, omics methods such as transcriptomics and quantitative proteomics are utilized to scrutinize the mechanisms of action of nanomedicine; however, these methods do not pinpoint the direct targets of nanomedicine. In addition to informing the design of carry‐based or carry‐free nanomedicines, emerging target discovery techniques can offer insights into the mechanisms of action of constructed nanomedicines. To investigate the anti‐tumor effects of the self‐assembled celastrol‐erianin nanomedicine (CEN), Tian et al. employed ABPP‐based chemical proteomics technology in 4T1 cells to identify Annexin A2 as a significant target of free CEL. Building upon this information, the researchers subsequently demonstrated that CEN could similarly interact with Annexin A2 to induce cancer cell apoptosis, thereby enhancing its inhibitory effect on breast cancer [100]. This finding suggests that the target elucidation of free drugs by target discovery techniques can provide valuable ideas for analyzing the mechanism of action of nanomedicines. However, such a strategy is not straightforward. Through passive or active targeting mechanisms, CEL nanomedicines can accumulate specifically at the disease site in vivo, rather than dispersing widely across various organs and tissues. This presents an opportunity to investigate the mechanisms of action of nanomedicines directly in vivo using target discovery approaches. For instance, chemical proteomics technology can be introduced into nano‐drug delivery studies by replacing CEL in nanomedicines with CEL probes (CEL‐P). Diseased tissues are isolated after nanomedicine injection, and the target proteins that bind to CEL‐P are pulled down and identified by MS after a click chemistry reaction, thus enabling direct elucidation of how CEL exerts pharmacological effects at the tissue level. Thermal proteome profiling (TPP, also known as MS‐CETSA) is a proteome‐wide label‐free target discovery method based on the principle that drug binding to target proteins can enhance their thermal stability. This method eliminates the need for drug probes and has found widespread application in target discovery of drugs [4]. The advancement of tissue‐level TPP represents a crucial avenue for future TPP development [102]. Following the accumulation of nanomedicines in vivo to elicit the drug's effect, the isolated diseased tissues can be subjected to transient gradient thermal challenge, and subsequent MS detection can identify target proteins exhibiting enhanced thermal stability. The employment of TPP not only detects the on‐target effects of drugs released from nanomedicines but also reveals the effects induced by nanocarriers simultaneously, making it a more suitable approach for investigating the mechanisms of action of nanomedicines (Figure 8D). By utilizing TPP‐based target identification workflow, Luo et al. demonstrated a direct interaction between self‐assembled nanomicelles of oleanolic acid and 20S proteasome subunit alpha 6 (PSMA6) in cancer cells, which suggested that PSMA6 served as a direct target for the nanomicelles in the induction of cancer cell pyroptosis [103]. This provides a successful example of the application of target discovery technology in elucidating the mechanisms of action for both carrier‐based and carrier‐free nanomedicines. Collectively, exploring the cross‐fertilization of target discovery and drug delivery research may bring new development opportunities in the field of drug delivery.

## 9 Discussion, Conclusion, and Future Perspectives

**FIGURE 9:**
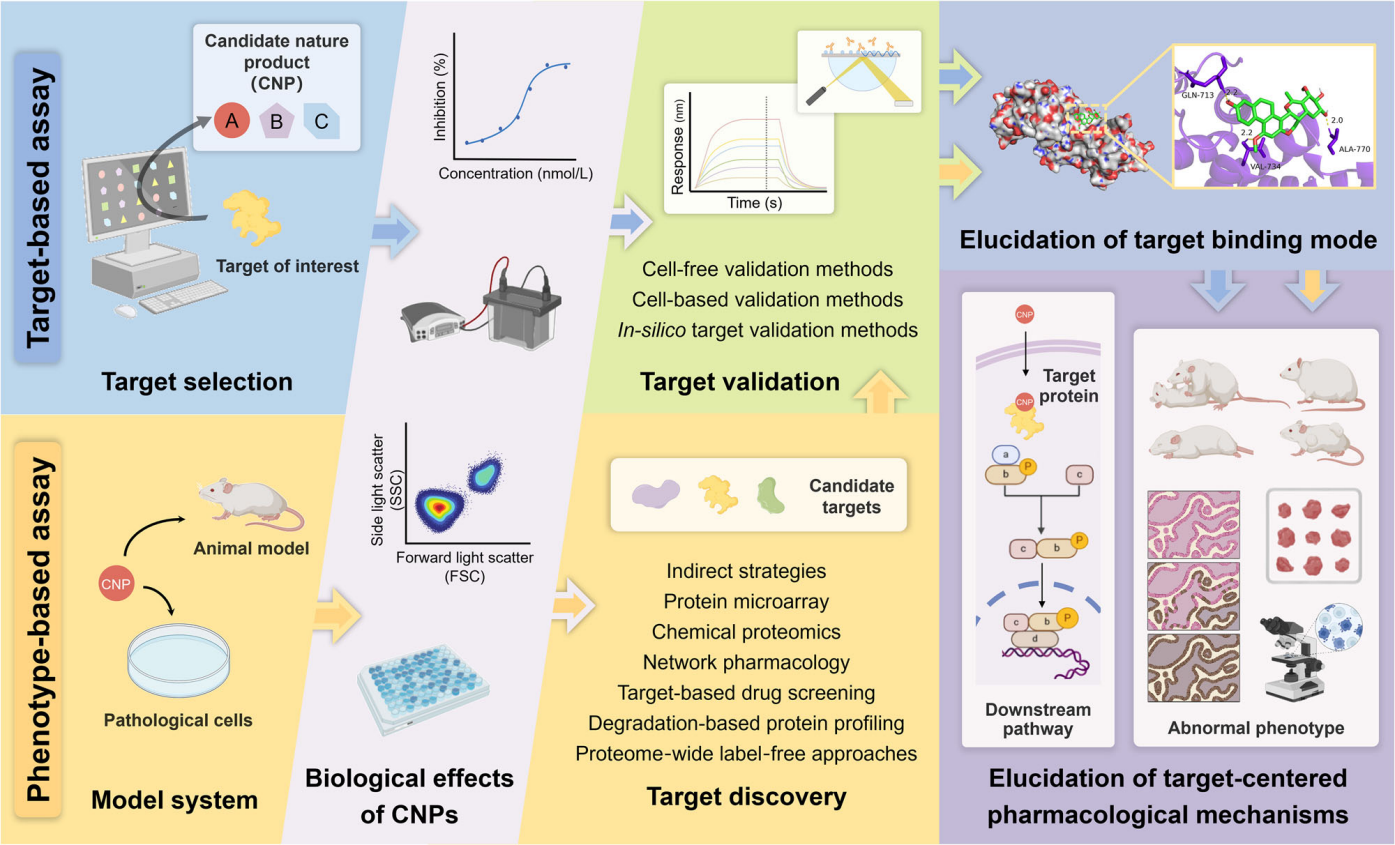
Schematic diagram of studies on target‐centered pharmacological mechanisms of natural products. CNP, candidate natural products.

CEL is currently considered a highly promising natural product for potential conversion into modern drugs. To achieve this goal, it is essential to comprehensively elucidate the targets of CEL as well as the target‐based mechanisms of action or toxicity. While hypothesis‐driven target confirmation approaches remain significant, thanks to the rapid development of MS technology, chemical biology, protein microarrays, and other relevant technologies, target identification of NPs has been expanded from low‐throughput target confirmation studies to the high‐throughput unbiased target screening at the proteomic level, such as chemical proteomics, proteome‐wide label‐free approaches, DBPP and protein microarrays. Moreover, multi‐omics‐based indirect strategies and computational methods, such as network pharmacology and virtual docking, also play important roles in the target identification of NPs. These strategies have been widely used in the target discovery of CEL with remarkable success. Among them, ABPP‐based chemical proteomics is currently the most favored method for CEL target identification, which may be largely attributed to the establishment of bioorthogonal probes administered at the carboxyl site of the CEL structure. The protein microarray strategy has further expanded the direct target discovery capabilities for CEL on a larger scale without being limited by specific pathological conditions. However, both chemical proteomics and protein microarrays necessitate chemical modifications on the NP molecule, potentially impacting the NP's activity and leading to inaccuracies in target identification. While label‐free approaches offer a way to investigate CEL targets without altering its activity, studies on CEL target discovery based on these approaches remain limited. Therefore, enhanced target identification studies based on label‐free approaches could provide strong support for the comprehensive elucidation of CEL target information. Since multi‐omics analysis strategies or computational methods are based on indirect experimental results or predicted results, sufficient evidence needs to be provided in subsequent target validation to support the conclusions. Furthermore, novel target discovery approaches such as genomic library screening [104], pH‐dependent protein precipitation (pHDPP) [105], thermostability‐assisted limited proteolysis‐coupled mass spectrometry (TALiP‐MS) [106], and single‐cell omics [15b] have emerged recently, offering a valuable supplement to the comprehensive interpretation of CEL target information. In addition, the growing volume of biomedical big data underscores the potential of AI‐driven target prediction methods for the discovery of disease and drug targets [107]. Larger scale, higher throughput, and more accurate target discovery of NPs represented by CEL depends on the advancement and cross‐fertilization of multiple disciplines, such as biology, chemistry, medicine, and AI, which provides unlimited possibilities for future drug‐target studies. Any target discovery strategy inevitably suffers from false positive results and incorrect identification. Although there is no “right answer” as to which method is best, the precision of identifying CEL targets can potentially be improved by integrating multiple target discovery approaches along with setting rigorous validation criteria for target binding. In addition, after target discovery, multi‐omics analysis can be combined to reveal the indirect network perturbations caused by NPs‐target interactions, which is very important for sorting out the clues of the mechanisms of action. The target discovery and target‐centered pharmacological mechanism research strategy for NPs represented by CEL can be seen in Figure 9.

The emergence of target discovery approaches in recent years has significantly contributed to the elucidation of how CEL plays therapeutic roles in different diseases, which greatly promotes the development of CEL‐based drugs. It should be noted, however, that dozens of targets of CEL have been identified using these approaches, and present the phenomenon of different targets of action in different disease contexts. These findings underscore CEL's status as a multi‐target drug. In contrast to single‐target drugs, multi‐targeted molecules possess the capability to interact with multiple biological targets concurrently, thereby facilitating a more comprehensive regulation of biological pathways, particularly in the context of complex diseases such as cancer and diabetes. This multi‐target approach addresses the limitations associated with the efficacy of single‐target therapies. Furthermore, there is potential for synergistic interactions among various targets, enabling multi‐target molecules to elicit enhanced therapeutic outcomes through the simultaneous modulation of multiple pathways. Through multi‐target design, molecules may spread out their effects among different targets, thus reducing the strong effect on a particular target, and thus reducing side effects. Additionally, these multi‐targeted agents may enhance their bioavailability by improving the absorption and distribution of the drug via diverse mechanisms. Moreover, by engaging multiple targets at once, multi‐target molecules can diminish the likelihood of developing drug resistance. Given these advantages, there has been a growing interest in the design and development of multi‐targeted drugs in recent years. Natural products have consistently demonstrated a distinct advantage in the realm of multi‐target drug development [108]. However, in the pharmacological studies after target discovery, the majority of studies have focused on a single target associated with a certain disease, which raises concerns about whether this can fully reveal the underlying mechanisms related to the pharmacological or toxic effects of CEL. While a specific target may be recognized as a therapeutic target for CEL in a particular disease context, its potential role as an off‐target or toxic target in another disease state remains challenging to ascertain and has not received sufficient attention. Furthermore, beyond therapeutic benefits, the biological toxicity of CEL, including hepatotoxicity and reproductive toxicity, must not be overlooked. Presently, CEL target research predominantly emphasizes therapeutic targets, with limited exploration of off‐targets or toxic targets. Therefore, enhancing the comprehensive target profile of CEL, establishing robust methodologies for evaluating reported target data, and systematically delineating the interplay between CEL targets, therapeutic outcomes, side effects, and toxicities represent crucial avenues for future research. In addition, considering that the contribution of each target to CEL in disease treatment is unequal, there is a need to focus on the role of CEL in the modulation of key targets such as PRDXs, HMGB1, HSP90, STAT3, and PKM2. This emphasis is particularly crucial for the guidance of future structure optimization of CEL.

Although extensive research has been conducted on CEL in preclinical studies, the development of CEL‐based drugs encounters significant obstacles. Challenges such as low water solubility, inadequate bioavailability, and potential off‐target toxicity of CEL severely restrict its clinical applications [8a]. Rapidly evolving target discovery methods have opened a window for monitoring off‐target effects and side effects of CEL in a clinical setting, which provides effective guidance for rational clinical dosing of CEL. In addition, the introduction of combination therapies, carrier‐free or carrier‐based nano‐delivery systems presents extensive potential to enhance the clinical utilization of CEL. The target information of CEL provides effective guidance for the rational design of combination therapies and nano‐drug delivery systems, especially when designing the co‐administration of CEL with drugs of differing mechanisms of action. In addition, introducing emerging target discovery methods into drug delivery research may also provide a solution to elucidate the mechanisms of action of nanomedicines. In addition, there is a growing interest in modifying the CEL chemical structure to generate more effective and less toxic derivatives or analogs [9, 109]. Previous structural optimization of CEL has mostly been performed under the guidance of phenotype‐based bioactivity evaluation of CEL derivatives. The identification of CEL targets offers the opportunity for structural optimization guided by the effect of CEL on specific targets, that is, based on the discovery of specific targets of CEL associated with specific activities, the inhibition or activation of the target protein is used as an assessment criterion to develop new, highly selective, and effective target inhibitor drugs, particularly covalent inhibitors.

In summary, this review provides a comprehensive overview of different approaches to identifying targets for CEL, along with the associated target information. It further discusses the relationship between CEL targets and their therapeutic effects and side effects, underscoring the significance of clarifying target profiles for the enhancement of CEL clinical practice. The review notably emphasizes the active role of target discovery in promoting CEL‐based combination therapies and drug delivery, which is essential for the further advancement of clinical applications of CEL. This review aims to elucidate the scientific connotation of the diverse pharmacological effects of CEL by using the target as a link and to provide a theoretical foundation for the drug development of CEL‐based drugs.

## Conflicts of Interest

The authors declare no conflicts of interest. Meiwan Chen is a member of the *Exploration* editorial board and she was not involved in the handling or peer review process of this manuscript.
